# Genome analysis of triple phages that curtails MDR *E. coli* with ML based host receptor prediction and its evaluation

**DOI:** 10.1038/s41598-023-49880-x

**Published:** 2023-12-27

**Authors:** Vineetha K Unnikrishnan, Niranjana Sri Sundaramoorthy, Veena G. Nair, Kavi Bharathi Ramaiah, Jean Sophy Roy, Malarvizhi Rajendran, Sneha Srinath, Santhosh Kumar, Prakash Sankaran S, Suma Mohan S, Saisubramanian Nagarajan

**Affiliations:** 1 Center for Research On Infectious Diseases (CRID), School of Chemical and Biotechnology, SASTRA Deemed University, Thanjavur, Tamil Nadu 613401 India; 2 Translational Health Sciences Technology Institute, Faridabad, India; 3 Department of Bioinformatics, School of Chemical and Biotechnology, SASTRA Deemed University, Thanjavur, Tamil Nadu 613401 India; 4 Antimicrobial Resistance Lab, ASK-I-312, School of Chemical and Biotechnology, SASTRA Deemed University, Thanjavur, Tamil Nadu India

**Keywords:** Applied microbiology, Bacteriophages, Computational biology and bioinformatics

## Abstract

Infections by multidrug resistant bacteria (MDR) are becoming increasingly difficult to treat and alternative approaches like phage therapy, which is unhindered by drug resistance, are urgently needed to tackle MDR bacterial infections. During phage therapy phage cocktails targeting different receptors are likely to be more effective than monophages. In the present study, phages targeting carbapenem resistant clinical isolate of *E. coli* U1007 was isolated from Ganges River (U1G), Cooum River (CR) and Hospital waste water (M). Capsid architecture discerned using TEM identified the phage families as *Podoviridae* for U1G, *Myoviridae* for CR and *Siphoviridae* for M phage. Genome sequencing showed the phage genomes varied in size U1G (73,275 bp) CR (45,236 bp) and M (45,294 bp). All three genomes lacked genes encoding tRNA sequence, antibiotic resistant or virulent genes. A machine learning (ML) based multi-class classification model using Random Forest, Logistic Regression, and Decision Tree were employed to predict the host receptor targeted by receptor binding protein of all 3 phages and the best performing algorithm Random Forest predicted LPS O antigen, LamB or OmpC for U1G; FhuA, OmpC for CR phage; and FhuA, LamB, TonB or OmpF for the M phage. OmpC was validated as receptor for U1G by physiological experiments. In vivo intramuscular infection study in zebrafish showed that cocktail of dual phages (U1G + M) along with colsitin resulted in a significant 3.5 log decline in cell counts. Our study highlights the potential of ML tool to predict host receptor and proves the utility of phage cocktail to restrict *E. coli* U1007 in vivo.

## Introduction

Drug resistant microbes have become more widespread in the environment globally which could be partially attributed to antibiotic misuse/overuse, coupled with evolutionary selection pressures^1,2^. Interestingly recent studies show that even non antibiotic compounds like microplastics, metal nanoparticles and non antibiotic drugs could also increase the prevalence of antimicrobial resistant genes in the environment^3^. The issue of antimicrobial resistance gets further exacerbated due to horizontal gene transfer among microbes in the environment^4^. These factors have made many antimicrobials obsolete leaving just higher generation antimicrobials like cephalosporins(4th and 5th generation), carbapenems, fosfomycin etc. and a handful of last resort antimicrobials like tigecycline, colistin, daptomycin, vancomycin and linezolid as effective lifesaving drugs. Unfortunately, both inherent and acquired resistance has been reported even for the last resort drugs^5^, threatening a shift to post antibiotic era as warned by WHO^6^. Given this dire situation, there is an urgent unmet need to find alternate solutions to curtail infections by drug resistant bacteria^7^. Among the alternative resistance modulatory approaches like betalactamase inhibitors^8,9^, efflux pump inhibitors^10,11^, phage therapy, a biological control measure, which involves use of lytic bacterial viruses to kill bacteria is considered as a viable alternative to prevent /treat bacterial infections. Phages are most diverse and wide spread in different environments and estimates show that roughly around 10^30^ phages prevail in the biosphere^12^. Phages can effectively mitigate both drug sensitive and drug resistant bacteria^13^. With the help of phage encoded depolymerases, they can efficiently target biofilms which are resilient to diverse antimicrobials^14,15^. In addition, phages can propagate in vivo and co-evolve to target phage-resistant bacterial strains^16,17^. Owing to these desirable features, phages qualify to be a tolerable, non-toxic and a reliable tool to restrict drug resistant bacteria via phage therapy.

Phage therapy has been is in vogue in Georgia, Poland and Russian Federation for almost a century. In Belgium, phages are used as Magistral therapy to treat patients when conventional therapy fails^18^. Eliavia Phage Therapy Center, Georgia supplies customized phage cocktails to treat patients across the globe, especially those who fails to respond to conventional antibiotic therapy^19^. Recently, FDA has approved the Clinical trials for the use phage cocktail WRAIR-PAM-CF1 to restrain *Pseudomonas aeruginosa* in chronically colonized CF patients^20,21^. Earlier during COVID pandemic in case of critically ill COVID patients, as a compassionate measure, FDA had accorded the approval to use Phagebank ™ therapy from Advanced Phage Therapeutics to curtail MDR strains of *A. baumannii*, *S. aureus* and *P.aeruginosa*^22^. Despite many successful clinical outcomes reported for personalized phage therapy^23–25^, phage therapy is yet to gain foothold as an active therapeutic practice, due to certain aspects like narrow specificity, shelf life and resistance/persistence of certain strains to phage therapy, which is attributed to adaptation/species diversification^26^.

Numerous research works^27^ have highlighted ability of phages from diverse sources to curtail clinically relevant MDR pathogens during experimental infections in animal models including *Clostridium difficile*^28^, Vancomycin resistant *Enterococcus faecium*^29^, Impepenem resistant *P. aeruginosa*^30^, MDR *S. aure*us^31^ and O25b: H4-ST131 *Escherichia coli* strain producing CTX-M-15^32^ and, Carbapenem resistant *Klebsiella pneumoniae*^33,34^ in mice models. Among the reports on phages targeting *E. coli*, lytic bacteriophage belonging to *Myoviridae* from urban sewage, vB_Eco4M-7, was shown to be effective against multiple *E. coli* O157 strains, and the phage did not harbour any toxins and virulence factors^35^. Similarly a phage PDX, belonging to *Myoviridae*, isolated from wastewater in Portland killed diarrhoeagenic enteroaggregative *E. coli* isolates, leaving the human microbiome undisturbed^36^. Multiple studies have reported advantage of using phage antibiotic combinations^37^ as the evolution of resistance to phages induces a fitness cost in host^38^. A recent report showed that the attempts by microbe to enhance ampicillin resistance by AmpC expression collaterally led to its susceptibility to phage, possibly by employing OmpA (outer membrane protein) as receptor^39^, which favors the concurrent use of antibiotics and phage therapy in synergy. Phage antibiotic synergy was reported earlier for *E.coli* phages wherein, a sub lethal doses of ciprofloxacin and ECA2 phage (*Podoviridae*) exhibited synergy against *E. coli*, causing 7.8 log CFU/ml decline in 8 h^40^. Studies have also highlighted advantage of phage cocktails over monophage therapy against highly refractory *K. pneumoniae* ST258 strain^41^ and against enteropathogenic *E. coli* serotypes 0157: H7 and 0104:H4^42^. Computational methods to predict bacterial host range are well reported^43,44^. But reports on attempts to predict host cell surface receptor using machine learning based approaches has been limited^45–47^.

In the present study, 3 different phages targeting MDR colistin heteroresistant clinical isolate of *E. coli* U1007, was isolated from water bodies (2 Rivers and a Hospital Waste Water) and the isolated phages were characterized. From the genome sequence of phage, the Receptor Binding Protein RBP (tail spike protein) was identified and by employing machine learning algorithms, host cell surface receptor was predicted, which was further validated by growing the host under conditions that is well known to alter the expression of some of these receptors. Furthermore, the ability of the isolated phage to curtail growth of MDR *E. coli* was explored with monophages and phage cocktail both in the presence and absence of colistin in vitro and in vivo.

## Results

### Identification of phages targeting MDR *E. coli* strains

Different sources viz., The Ganges River, The Cauvery River, Cooum River, Hospital waste water, pond samples, soil samples from farmland, samples from cow shed were collected and screened for presence of bacteriophages specific to carbapenem resistant and colistin heteroresistant MDR strain of *E. coli* (U1007). Only samples that displayed lytic activity against MDR *E. coli *U1007 strain were taken up further (Figure S1). Among the different water samples tested, we found that the water samples from The Ganges River (U1G), The Cooum River (CR) and Hospital waste water (M Phage) harbored lytic bacteriophages specific to the MDR *E. coli* U1007. As the other water samples did not possess lytic phages targeting MDR strain of *E. coli*, they were not pursued further. The Genome of *E. coli *U1007 strain was sequenced using illumina platform and sequence was submitted to NCBI Genbank (SRA Accession No: PRJNA988283). The AMR genes were identified using Resfinder, RAST and Roary and is presented in Table S1. Analysis of the antimicrobial resistant genes (ARG) in the genome of U1007 revealed that the strain harbored resistance to 48 antimicrobials belonging to multiple antimicrobial classes viz., cephalosporins, floroquinolones, macrolides, carbapenems, tetracyclines including efflux transporters that have been associated with colistin resistance (Table S1). Determination of Minimum inhibitory concentration (MIC) for U1007 showed that strain exhibited resistance to 8 different antimicrobial classes  hence it was deemed as an MDR strain (Table S2). The drug resistance profile of the other clinical isolates (U3790, and U2354) used in this study were reported earlier^48^.

### Isolation and purification of U1G, CR and M phages

**Figure 1:**
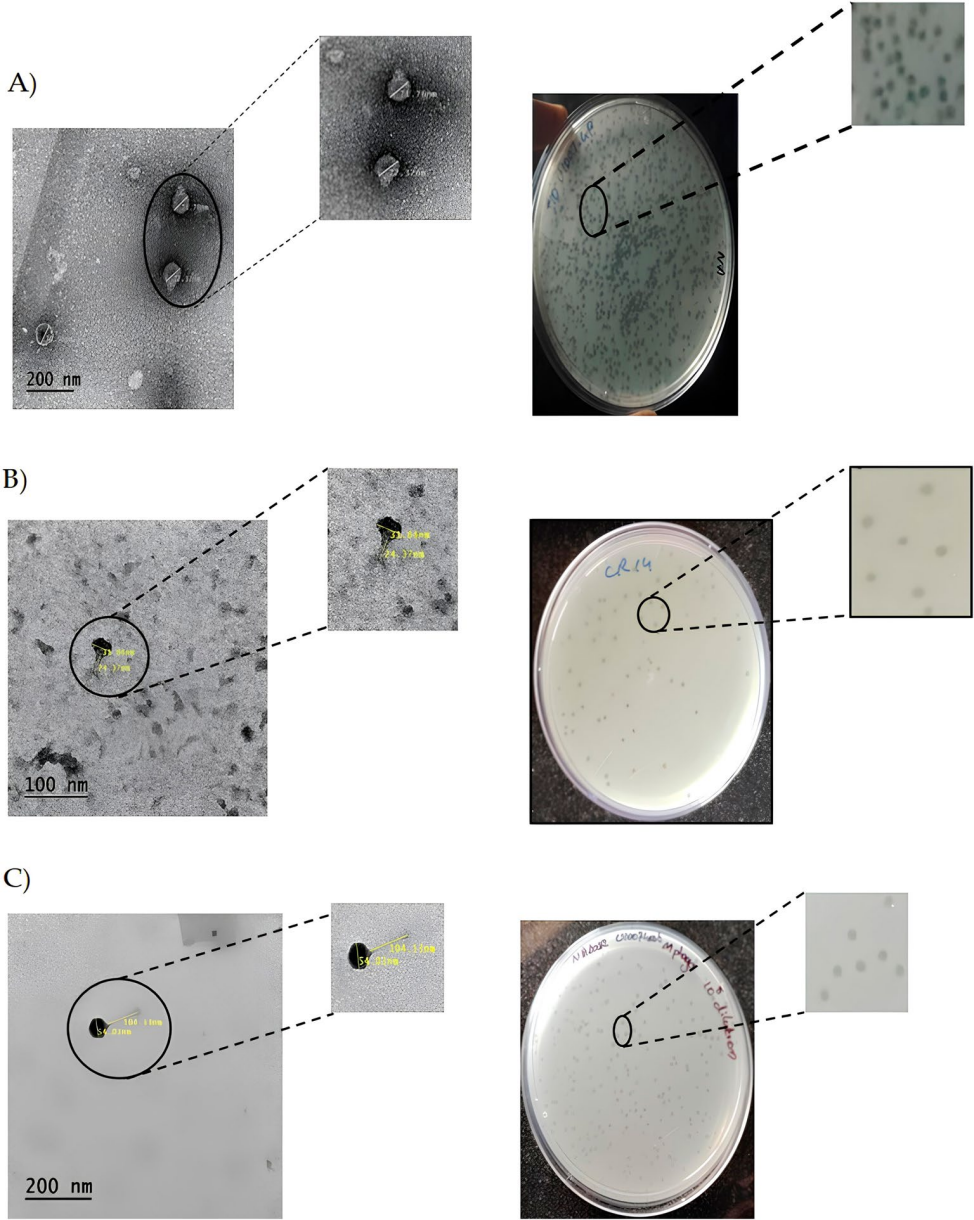
Phenotypic characterestics of the isolated phages : TEM images of the isolated phages (left panel) and plaque morphologies (right panel) (**A**) U1G, (**B**) CR phage and (**C**) M phage.

Spot assay revealed the presence of bacteriophages targeting U1007 strain from The Ganges river, Cooum River and Hospital waste water. In order to isolate these phages, the supernatant was filtered, serial diluted, incubated with U1007 for 20 min and overlaid on nutrient agar plates. Post incubation, the plaques were identified based on clear zone of lysis. The phage morphology was carefully observed and a single plaque morphology was taken up for purification. The phages were triple purified ensuring that similar plaque morphology was repeatedly obtained (Fig. 1) and a phage stock of high titer (10^13^ PFU/mL) was stored at 4^o^C for further use. The phage specific to U1007 from Ganges was designated as U1G, Cooum river as CR and Hospital waste water as M phage respectively.

### TEM imaging

The morphology of phages were observed by staining with 2% uranyl acetate using Transmission electron microscopy. The TEM image showed that all 3 phages possessed an icosahedral head of varying diameter ranging from 71.76 nm for U1G, 31.64 nm for CR phage and 54.03 nm for the M phage. U1G had a very short non-contractile tail, whereas CR phage had a non-contractile tail of 24.37 nm and M phage had a long non-contractile tail of 104.13 nm (Fig. 1). Thus based on phage morphologies, as discerned by TEM, U1G belongs to *Podoviridae,* CR phage belongs to *Myoviridae* and M phage belongs to* Siphoviridae.*

### Genome analysis

**Figure 2:**
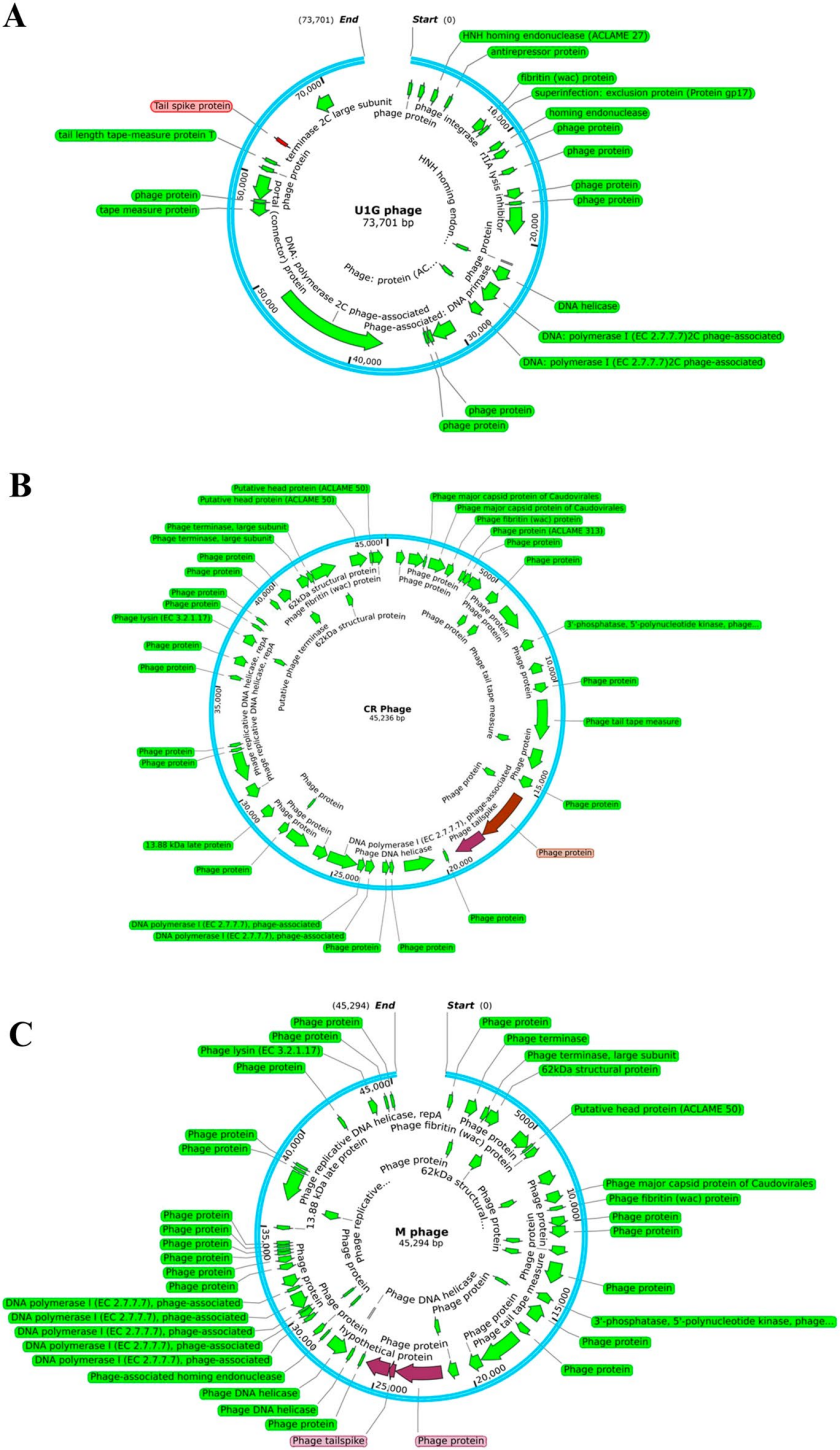
Genome maps of Escherichia phages (A) U1G, (B) CR Phage and (C) M phage. Putative ORFs of the genome excluding hypothetical proteins annotated using RAST are depicted. Tail spike protein (Receptor Binding Protein) is indicated in red in the genome map. The image was constructed using SnapGene6.2.

Whole genome sequencing of U1G, CR and M phages were performed using Oxford MinIon Nanopore platform. The raw reads of the U1G phage were trimmed and corrected using Porechop and assembled to contigs using Canu v 1.8. The assembled contigs were annotated using RAST and the genome sequence was submitted in NCBI GenBank (Accession Number: MZ394712). The genome of U1G is 73,275 bp long and it has a GC content of 43%, N50 of 73,275 and L50 as 1. The depth of coverage of the U1G genome was 39.12X. RAST annotation revealed the presence of 91 coding sequences, out of which 31 sequences code for phage packaging, replication, and other functions (Table S3). Rest of the proteins were annotated as hypothetical. Annotation of phage genome sequence by PHASTER revealed that the genome was intact. PhagePromoter tool predicted 54 promoters in the genome and absence of tRNAs were revealed by tRNAscan-SE. Genome map of U1G constructed using SnapGene 6.2 showed that the majority of the identified functional modules clustered in the first half of the genome (Fig. 2A).

The assembled contigs of CR phage and M phage were annotated using RAST and the genome sequence was submitted to NCBI GenBank Accession Numbers: (OR061068 & OR061069). The assembled genome of CR phage formed a circular genome with 45,236 bp with coverage of 248X. The maximum coverage for M phage was 21X. From the Flye assembly of M phage reads, the contig with maximum coverage alone considered for further analysis. The selected contig was a circular one with 45,294 bp long. The GC content of CR and M phages was found to be 50.9%. RAST annotation identified the presence of 95 and 104 features in CR and M phages, respectively (Tables S4 and S5) and both the genomes were marked by the absence of tRNAs as revealed by tRNAscan-SE. Genome map of CR and M phage is reported in Fig. 2B and 2C, respectively.

**Figure 3:**
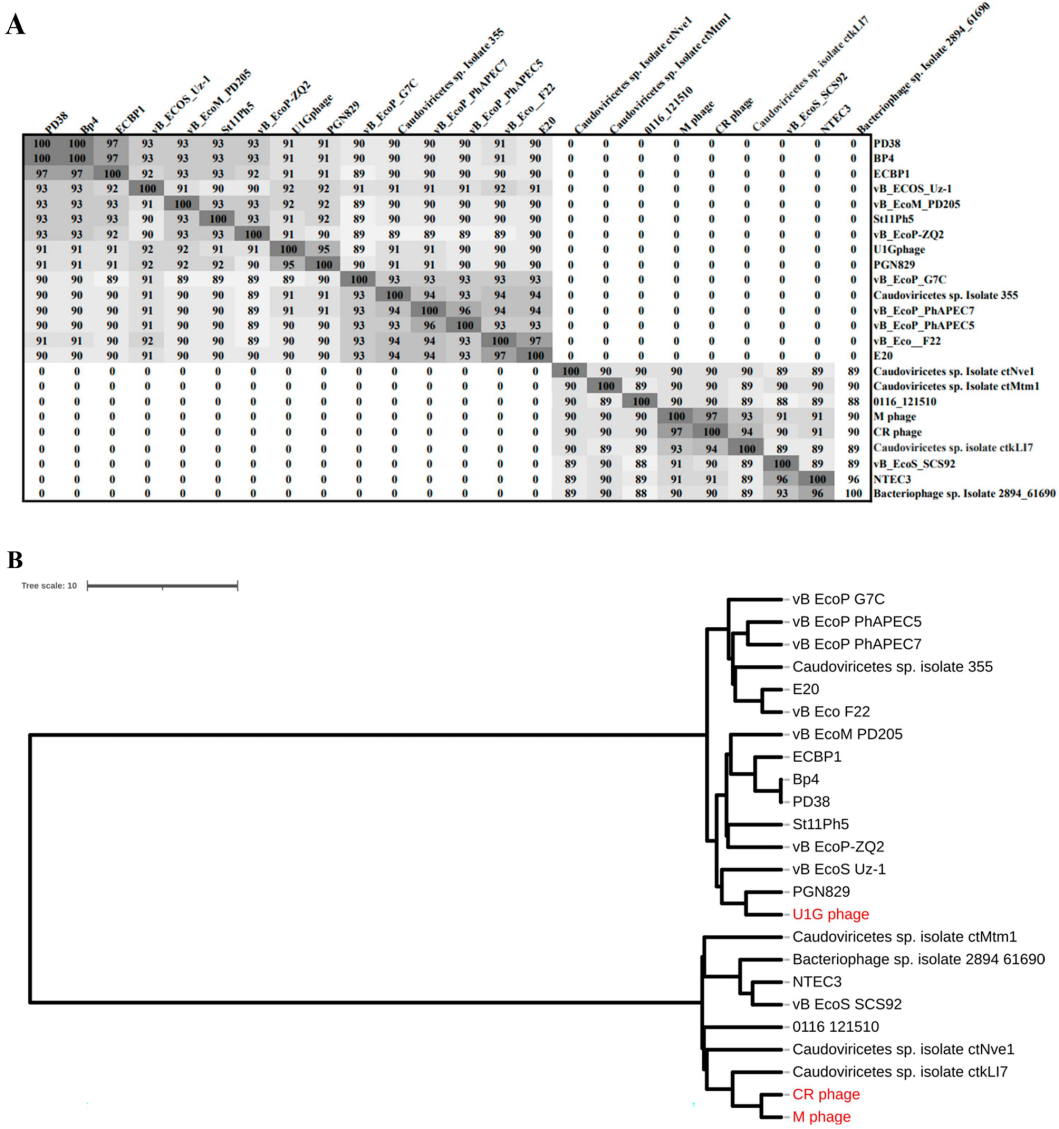
(**A**) ANI matrix calculated for the close homologs of Escherichia phages U1G, CR and M phages using ANI/AAI-Matrix tool (http://enve-omics.ce.gatech.edu/g-matrix/). (**B**) Phylogram constructed using the UPGMA clustering method from ANI/AAI-Matrix tool. CR and M phage are phylogenetically closely related and cluster together whereas U1G phage clusters separately. Visualization is performedusing iTOL v6.

The top 14 close homologs of U1G phage genome from BLASTn search were, PGN829, vB_EcoS_Uz-1, vB_EcoM_PD205, PD38, Bp4, St11Ph5, vB_EcoP_PhAPEC7, vB_EcoP_PhAPEC5, vB_EcoP_PhAPEC7, vB_EcoP-ZQ2, *Caudoviricetes* sp. Isolate 355, vB_EcoP_G7C, vB_Eco_F22, ECBP1, and E20. The CR and M phages showed 97% similarity and there are seven close homologs identified for both phages and are *Caudoviricetes* sp. Isolate ctNve1, *Caudoviricetes* sp. Isolate ctMtm1, 0116_121510, *Caudoviricetes* sp. isolate ctkLI7, vB_EcoS_SCS92, NTEC3, and Bacteriophage sp. isolate 2894_61690. The close homologs of the three phages were used for the ANI matrix calculation and the construction of Phylogram (Fig. 3). The results indicate that the U1G phage is distinct from CR and M phages as they form two separate clusters. NCBI BLASTn results showed that the U1G genome matched 95.98% with *Escherichia* phage PGN829.1 with 90% query cover and e value 0.

Phylogenetic tree also revealed that U1G is a close relative to *Escherichia* phage PGN829.1 (Fig. 3B). However, PGN829.1 was classified under a new family *Schitoviridae*^49^, which is highly similar to *Podoviridae* in morphology but harbors virion associated RNA polymerase. PHASTER showed the presence of RNA polymerase subunit in the U1G genome. BLAST analysis performed to detect the presence of virion associated RNA polymerase gene with U1G revealed the presence of homologous region (45,314–48,093 bp). Thus U1G was also classified as *Schitoviridae* although the presence of RNA polymerase in the U1G genome was not detected by RAST annotation. Mauve based comparative genome analysis of the close homologs of U1G (identified using ANI matrix) revealed three homologous blocks shared among the genomes. The homologous blocks are syntenic but positional variation for these syntenic regions was observed in different phages (Figure S2A) implying that these phages have a common ancestry. Genome alignment of CR and M phages along with their three close homologs revealed the presence of four homologs blocks shared among the genomes (Figure S2B). The CR and M phages showed highest similarity with *Caudoviricetes* sp. isolate ctkLI7 (Fig. 3).

The Potential RBP sequences of the U1G, CR and M phages were identified by using local BlastP search of the RAST annotated protein sequences in the phage genome with the RBP database(﻿Supplementary File 2)^46^. The details of the predicted RBPs in the bacteriophages and their close homologs in RBP database are reported in Table S6. RBPs are highlighted in the genome maps of U1G, CR and M phages reported in Fig. 2. The phage tail spike protein with 107 amino acid sequence is recognised as the RBP encoded by U1G phage which is used by the phage to attach to bacterial cell surface. The tail spike protein of U1G phage showed 85% similarity with the tail fibers protein of *Salmonella* phage SP1 of RBP database with an e-value of 4e-34 (Table S6). The homologs of the tailspike protein of U1G from other bacteriophages were obtained using web BLASTp search and MEGA11 was used to construct the phylogenetic tree of tailspike protein using UPGMA method with 1000 bootstrap values. The results revealed that tailspike protein of U1G was a close homolog of PGN829 hypothetical protein and is distantly related to tail fiber protein of *Salmonella* phage SPHG3 (Figure S3A). The hypothetical protein of PGN829 with Accession no. AXY82585 is a lengthy one with 628 amino acids. The tail fiber domain-containing protein of *Shigella* phage pSb-1 (119 amino acids) and the tail spike protein of Dompiswa phage TSP7_1 (102 amino acids) are the RBPs with length comparable to that of tailspike protein of U1G (Figure S3A).

There were two potential RBPs identified from the CR phage which were highlighted in the genome map in Fig. 2B. and reported in Table S6. The first one has 828 amino acids and is annotated as phage protein which showed 99% similarity with putative tail protein of* Escherichia *phage ST2 in the RBP database with an e-value of 0. The second one has 481 amino acids and is annotated as phage tailspike protein by RAST which showed 81% similarity with the tailspike protein of *Salmonella* phage FSL SP-049 with an e-value of 9e-69. The tail protein from the *Caudoviricetes* sp. identified as the close homolog of the first RBP from the CR phage using web BlastP search (Figure S3B). Tailspike protein from *Caudoviricetes* sp. and *Escherichia coli* phage are identified as the close homologous sequences of second RBP sequence of CR phage from Blastp search (Figure S3B).

There were three potential RBPs identified from the M phage which includes a phage protein, phage tailspike protein and a hypothetical protein (Fig. 2C, Table S6) with lengths 852, 110 and 437 aa respectively by search against the RBP database. Putative tail protein of *Escherichia* phage ST2, tailspike protein of *Salmonella* phage FSL SP-049 and tail fiber protein_*Acinetobacter* phage Petty from RBP database showed 99%, 86% and 45% similarities(with e-values 0, 1e-56 and 1e-16, respectively) with the three potential RBPs from M phage (The first two are reported in Table S7 and Figure S3C). Tail protein from Escherichia phage ST20 identified as the close homolog of the first RBP of M phage using web BlastP search. The tailspike protein from *Caudoviricetes* sp. identified as the close homolog of the second and third RBPs from M phage which are the tail spike protein and the hypothetical protein (Figure S3 C).The predicted RBPs present in the U1G, CR and M phages showed significant similarity with the curated entries in the RBP database and can be considered as the factors contributing to bacteriophage host recognition. Therefore, the top hits of the predicted RBPs from U1G, CR and M phages were used for the identification host receptor using machine learning approaches.

### A multiclass-classification model for bacteriophage host-receptor prediction in *E. coli*

ML based multi-class classifier using phage RBP sequence was created to predict the potential host receptors for *Escherichia* phage U1G. The RBP host receptor dataset for *E. coli* targeting phage with 160 entries were obtained from the following sources viz., report by Boeckaerts, D. et al.^46^, PhReD database and literature survey (Supplementary File 2). The 218 nucleotide and protein sequence features of the collected RBP sequences were obtained using the script reported earlier^46^ and were used for training the ML classification algorithms. The host receptors available in the dataset includes, LPS, Tsx, OmpC, OmpA, LPS core, FhuA, LPS O antigen, LamB, OmpF, FadL, TonB, BtuB, and pili tips.The distribution details of these receptors are reported in Figure S4. Three Classification algorithms, Random Forest, Multinomial Logistic Regression and Decision Tree were used for the construction of the multiclass classification models which can predict the host receptors of *E. coli* targeted by phage based on the Receptor Binding Protein (RBP) sequence of the bacteriophage. Multinomial Logistic Regression^50^utilizes linear combinations of features to model class probabilities making it well suitable for multiclass classification tasks under the assumption of linearity. In contrast, both Random Forest^51^ and Decision Tree^52^ excel in capturing non-linear relationships. Decision trees iteratively split the dataset based on the most informative features, while Random Forest, an ensemble of multiple decision trees effectively handles complex data patterns.

Performance comparison of the classification algorithms is reported in Table 1 and the model using the Random Forest Classifier Algorithm reported the best Performance in terms of all the metrics followed by the Multinomial Logistic regression Model. We have applied two feature selection methods, ANOVA and L2 Regularization and the top selected 30 features from the first and 110 features from the second method were used for the construction of feature selected ML models and the results are included in (Table 1). Overall, the ML model using Random Forest Classifier on the data set consisting of all the 218 features selected produced the highest performance of 93% in terms of precision, 90% accuracy and an average AUC value of 0.99 in terms of individual class contributions aggregate.

**Table 1:** Overall Performance Metrics of Machine Learning tools.

Performance Score	All features			ANOVA feature selected Dataset			L2 Regularization FEATURE Selected dataset		
	RF	DT	LR	RF	DT	LR	RF	DT	LR
Precision	0.93	0.88	0.91	0.86	0.85	0.85	0.84	0.64	0.78
Recall	0.90	0.86	0.91	0.86	0.83	0.83	0.79	0.66	0.77
Accuracy	0.90	0.84	0.90	0.86	0.83	0.83	0.79	0.66	0.77
F-1 Score	0.89	0.85	0.89	0.85	0.83	0.83	0.78	0.63	0.75
MCC	0.89	0.83	0.89	0.85	0.82	0.82	0.78	0.64	0.76

MCC- Matthew’s Correlation coefficient.

The predicted RBPs from the U1G, CR and M phages displaying high sequence similarity with the RBP database entries were used for predicting the host receptor using the multiclass-classification model constructed. The tailspike protein from U1G phage and phage protein from CR phage and M phage were used for the host receptor prediction. The host receptor prediction results from the Random Forest multi-class classifier using all features and selected features from ANOVA and L2 Regularization methods are reported in Table 2. For the U1G phage, LPS O antigen, OmpC or LamB were predicted as the host receptors. FhuA or OmpC were the predicted receptors for CR phage and OmpC, LamB, FhuA, TonB or OmpF were the potential host receptors for M phage (Table 2).

**Table 2:** Predicted Receptors for RBPs of U1G, CR and M phages using Random Forest based class classification method. Host receptors predicted using all features and the features based on ANOVA and L2 Regularization are included. The Receptor Binding Protein (RBP) from U1G, CR and M phages showing high similarity with the RBP database entries were exclusively included.

Phage	RBP	Predicted receptors		
		All Features	ANOVA	L2 Regularization
U1G phage	Phage tail spike protein (107 aa)	LPS O antigen	LPS O antigen	OmpC or LamB
CR phage	Phage protein (828 aa)	FhuA	FhuA	OmpC
M phage	Phage protein (852 aa)	OmpC or LamB	FhuA and TonB	OmpF

### Physiological validation of host OmpC as the receptor for U1G phage

**Figure 4:**
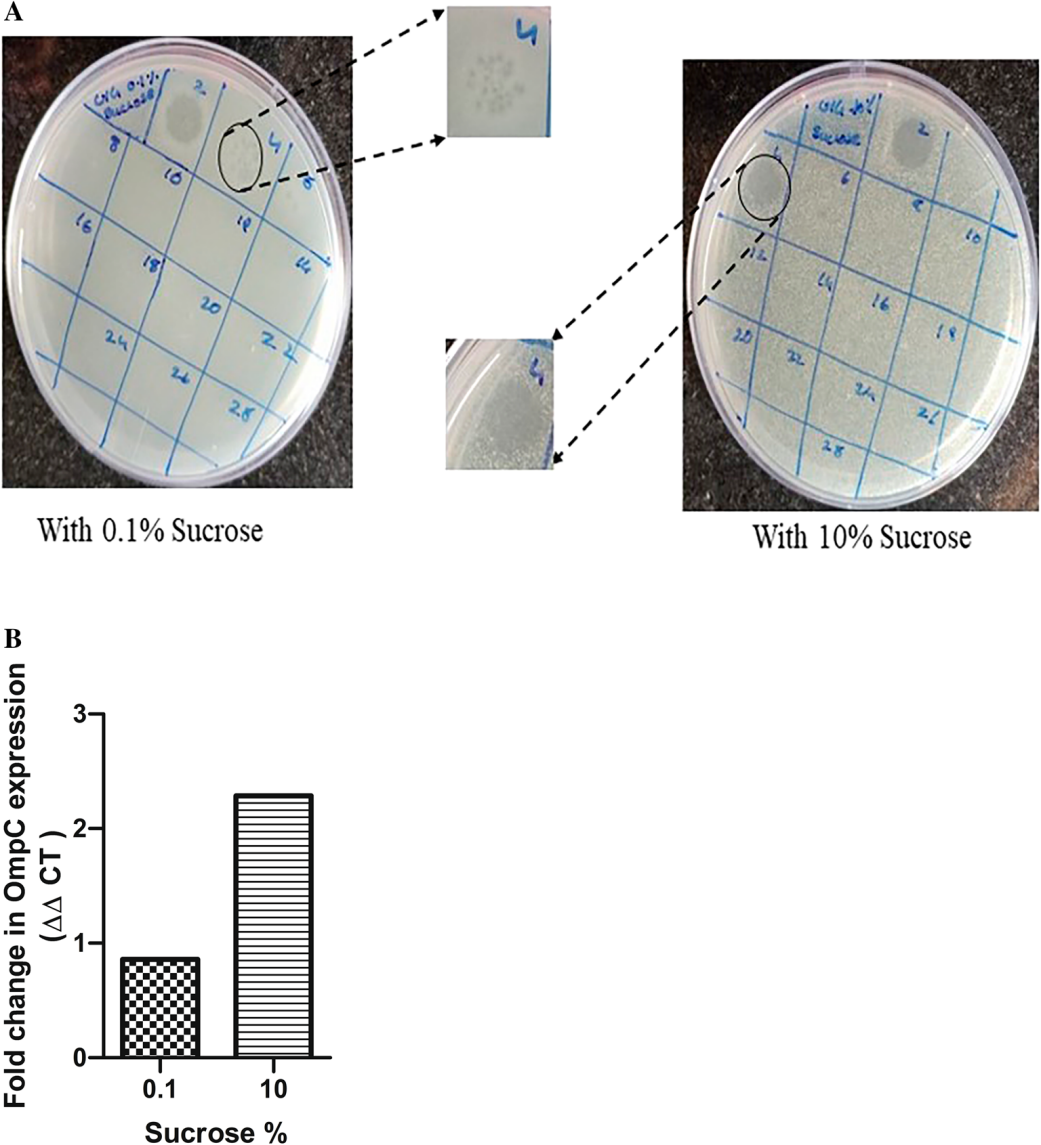
U1G phage employs OmpC as the host receptor for phage entry as predicted by RF algorithm. (**A**) growth under high osmolality conditions (10% sucrose) relative to low osmolality (0.1% sucrose) causes increased phage titers (**B**) Increased OmpC expression induced by growth under 10% sucrose relative to growth under 0.1% sucrose.

As RF algorithm using L2 regularization identified OmpC as one of the plausible host cell entry receptor for U1G, we checked whether differential expression of OmpC will affect U1G plaque titers, when the phage to host bacterial ratio was maintained constant. Based on the earlier well established reports^53,54^ on increased medium osmolality leading to differential expression of OmpC, we increased the medium osmolaltity using 10% sucrose in LB-NaCl medium and evaluated impact of increased OmpC overexpression on the phage titers relative to same medium containing 0.1% sucrose. As expected, U1G phages at 10^4^ dilution when mixed with 0.4 OD (A600) of U1007 *E. coli* raised in low osmolality medium (0.1% sucrose in LB-NaCl) had countable plaques whereas, the same dilution of phages (10^4^) when mixed with equivalent cell density [0.4 OD (A600)] of U1007 *E. coli* grown in high osmolality medium (10% sucrose in LB-NaCl) completely lysed the bacteria, resulting in total clearance proving high phage titers (Fig. 4a). Inorder to confirm the differential expression of OmpC, qRT-PCR was carried out in *E. coli* U1007 cells grown in LB-NaCl medium under 10% sucrose relative to cells grown in same medium with 0.1% sucrose. The results (Fig. 4b) revealed that high sucrose significantly upregulates OmpC expression as previously reported. Thus, increased OmpC expression (induced by high osmolality) most likely led to elevated levels of phage-host interactions which in turn results in enhancement in the number of host cells lysed relative to cells exhibiting relatively lower OmpC expression (caused by growth under low osmolality). Thus OmpC, as predicted by the ML tools, is a potential host cell surface receptor employed by U1G for entry into host cells. This observation would be further validated using OmpC knock out strain in an isogenic background in future studies.

### Burst size, latent period and host specificity

One-step growth curve showed that U1G possessed a relatively short latent period of 20 min, a rise period of 20 min and a burst size of 124 PFU/cell. M phage also had a short latent period of 20 min and a burst size of 150 PFU/cell. Whereas CR phage had a short latent phase of 15 min and a moderate burst size of 117 PFU/cell (Figure S5). The host range of U1G/CR/M phages were determined by testing the lytic activity of phage against different *E. coli* clinical isolates U3790, U1007, U3176, IDH09733, U2354, U1024, IDH09519, and MG1655, *Klebsiella pneumoniae* and *Salmonella* Typhimurium (since the tail spike protein of *Salmonella* phage showed higher sequence similarity with the tail spike protein of U1G phage) by spot assay. The results showed that all 3 phages U1G (Figure S6a) /CR (Figure S6b) /M (Figure S6c) showed tropism primarily towards U1007 and mild lytic activity against U2354, but not against other isolates. A faint lysis zone was exhibited by U1G against U3790 as the lysis zone was mild for U2354 and U3790, it was not explored further. Interestingly, ANI matrix based phylogenetic genome analysis of three *E. coli* clinical isolates U1007, U2354 and U3790 show that they cluster together with > 97 percent genome similarity and exhibits good similarity with genomes of Enterroaggregative *E. coli* (Figure S7). Ability of all three phages from diverse sources to exhibit lytic ability against U1007 and a faint lytic potential againt U2354/U3790 imply that host genome similarity could also be used to predict the host range of phages.

### pH and temperature sensitivity

Temperature and pH stability of U1G /CR/M phages were studied by incubating at different temperatures respectively. U1G remained stable in the temperature range of 4 °C to 45 °C and at 65 °C, 50% loss in viability was observed (Figure S7). Whereas the other two phages CR and M were stable only until 45 °C and both of them lost their viability when incubated at 65 °C with CR phage being more sensitive and quickly lost viability at 65 °C than M phage (Figure S8). The results of pH sensitivity revealed that U1G was stable at pH 5.0, 7.0 and 9.0 and at extreme pH of 3.0 and 11.0, U1G lost its viability (Figure S9). Altered ompC expression at extremes of pH might account for negligible lytic activity exhibited by U1G phage. CR phage was also inactived at pH 3.0 but relative to U1G phage, it retained 25% stability at pH 11.0 and M phage was stable until pH 9.0 and at pH 11.0 it retained 25% viability. Thus U1G had better thermotolerance whereas CR and M phages had better tolerance at alkaline pH. Thus all 3 phages differed in terms of their pH and Temperature stability which reinstates that although they target the same host, the three phages vary in their physicochemical characteristics.

### Time Kill Study

**Figure 5:**
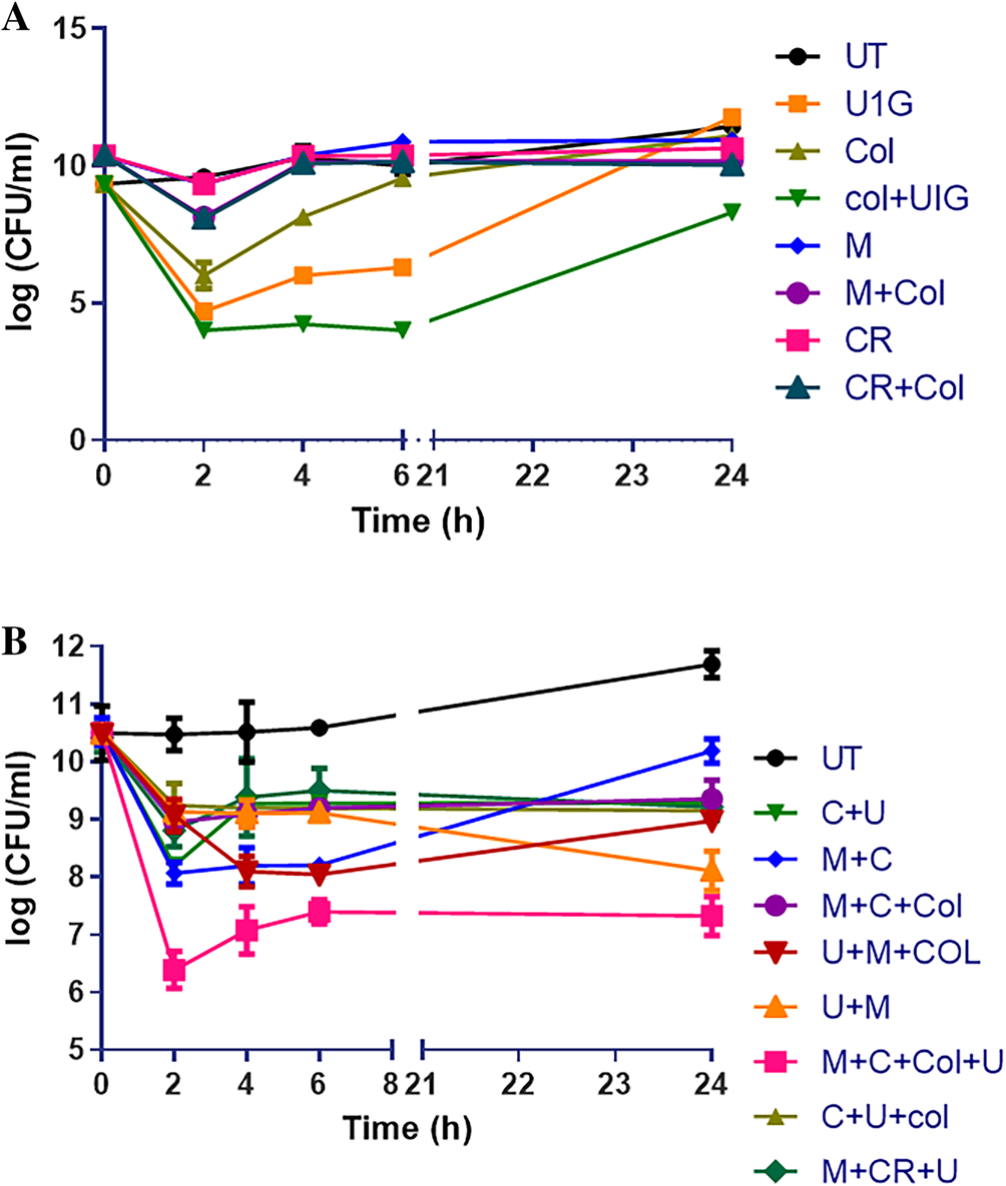
Time kill study. Time dependent kill analysis was performed by treatment of early log-phase cells of *E. coli* U1007 strain with **A**) monophages (U1G, CR and M) and **B**) phage combinations (U1G + CR, U1G + M, U1G + CR + M)- with and without colistin) and the samples from each group were retrieved at specific time points from 0– 24 h, serially diluted and plated on to LA plates and incubated at 37 °C. The colony count was expressed as log (CFU/ml). The experiment was performed in triplicates and the error bar represents their standard error of the mean. Student t test shows that triple phage combination with colistin was statistically significant relative to untreated control with a P value of 0.0039, similarly triphage along with colistin displayed significant variation relative to triphage cocktail without colistin (P = 0.052).

Time-kill was performed to evaluate the efficacy of Phage cocktails against U1007 individually and in combination with antibiotics. Our observations with monophages (U1G/CR/M) revealed that in a time kill experiment, monophages caused a decline in cell count only for the initial 2–3 h beyond which, almost a complete regrowth was observed by 24 h (Fig. 5A) which could be attributed to the probable clonal expansion of the resistant mutant. Treatment with phage combinations displayed a time kill trend similar to monophages but importantly, cell counts plateaued around 6 h and did not increase further even after 24 h, which shows that phage cocktails are highly effective in restricting regrowth relative to monophages. By 24 h, > 3 log decline in CFU was observed relative to founder population for triple phage combination along with colistin (U1G + M + CR + colistin) and a 2.5 log decline in cell counts were observed for phage combinations (U1G + M) relative to the initial CFU of the untreated control (Fig. 5B), these observations imply that phage cocktails are indeed effective in restricting bacterial growth. By 24 h, tri phage combinations with colistin had a 2.5 log decline in cell counts than phage cocktails without colistin, which implies that despite being highly effective, the tested phage cocktail might have additive effect but not synergistic effect with colistin in restricting U1007, as 3 log difference in CFU is usually regarded as synergestic.

As the strain displayed decline in cell counts due to colistin treatment in the time kill assay (Fig. 5), which was resembling trend reported earlier for colistin heteroresistant bacteria^55^, we evaluated whether the MDR clinical isolate of *E. coli* U1007 displays colistin heteroresistance by performing population analysis profile as reported earlier^56^. U1007 strain was serially diluted and spotted on plates containing increasing concentrations of colistin and cell counts were compared with strain plated on antibiotic free plates and if the ratio greater than 0.0001 the tested strain is deemed as colistin heteroresistant. Our calculations showed (Figure S10) that the ratio of cells grown on colistin (4 µg/ml) containing plates relative to colistin free plates is 0.00133 hence MDR U1007 strain in the present study was indeed colistin heteroresistant.

### In vivo toxicity and infection

As a representative of 3 phages, U1G phage was evaluated for its toxicity in zebrafish by estimating the brain and liver enzyme profiles. Different titers of phages (10^4^, 10^10^ and 10^12^ PFU/ml) were injected intramuscularly and the enzyme profiles were estimated. It was observed that there was a slight increase in α-naphthol release corresponding to control, but it was not statistically significant. The levels of β naphthol and acetylcholine esterase was similar to that of untreated control (Figure S11). Hence the phages are unlikely to pose any toxicity to zebrafish even at a relatively high doses.

**Figure 6:**
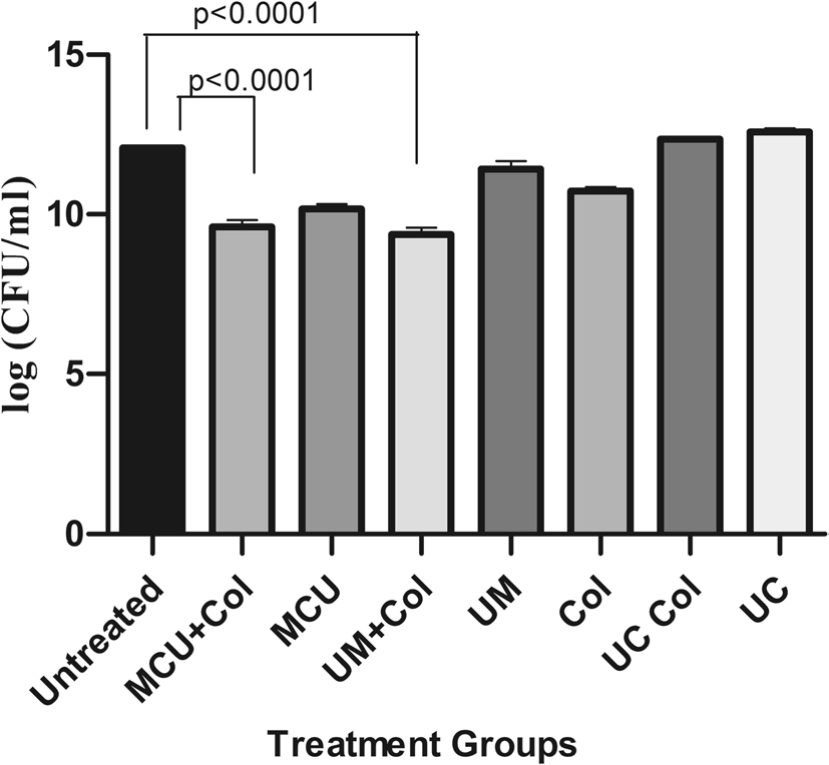
Phage cocktails (U1 + CR + M and U1 + M) along with colistin caused significant reduction in bioburden in infected zebrafish. Fish were infected with *E. coli* U1007 and 1 h post infection fish were grouped into untreated control and fish treated with bi-phage combination (U1 + M, U + C, M + C) and Tri-phage combination (U1 + M + CR) with and without colistin. 48 h after treatment, fish were euthanized, muscle tissues were dissected, macerated, serial diluted and plated onto LA plates. Plate counts were determined 24-48 h post incubation.. The experiment was performed in triplicates and the error bar represents their standard error of the mean. Student t test was performed to determine statistical significance of the variation among the treatment groups.

In vivo infection study was performed with monophages/phage combinations either alone or with sub MIC levels of colistin to restrict U1007 infection. The results showed that treatment with monophages resulted only upto 1.3 to 1.5 log reduction in bioburden whereas monophage + colistin treatments caused a maximum of upto ~ 2.1 log CFU decline relative to the untreated control. Colistin treatment alone caused 1.2 log decline in CFU (Figure S12). Phage combinations along with colistin especially bi phage (U1G + M) + Col resulted in 3.3 log decline in CFU and triphage (U1G + CR + M) + Col resulted in 3 log decline in cell counts (Fig. 6) which were statistically significant. These observations reiterates potential of phages and antibiotic combination to restrict growth of clinical isolate of *E. coli in viv*o. It is important to note that colistin exhibited synergy only with certain phage combinations and not with all phage combinations. In the bi-phage (U1G + M) combination treatments, relative to the untreated control, colistin exhibited synergy with the phage cocktail and caused 3.3 log decline in CFU relative to bi-phage (U1G + M) treatment alone, wherein, a meagre 0.8 log reduction in CFU was observed (Fig. 6). On the other hand, colistin did not exhibit synergy with triphage cocktail as both the triple phage (U1G + CR + M) and colistin + triple phage combination were comparable and only a modest decline of 0.5 log CFU was noted due to colistin addition to the triple phage cocktail. Hence ability of antibiotic to synergise with the phage is dependent on phage combination being employed.These results show that certain phage cocktails (U1G + M) along with colsitin is quite effective in restricting the bioburden of colistin heteroresistant MDR clinical isolate of *E.coli* in zebrafish infection model.

## Discussion

Bacteriophages have been reported against different pathogenic strains of *Pseudomonas aeruginosa, Clostridium difficile, Vibrio parahemolyticus, Staphylococcus aureus, Acinetobacter baumannii, E. coli and Klebsiella pneumoniae*, individually or in combination with antibiotics, thus favoring reuse of antibiotics^27^. In this study, we have isolated phages targeting MDR *E. coli* clinical isolate (U1007) that is resistant to multiple antibiotics, especially to drugs like carbapenems and is also ESBL positive and hence it falls under the critical priority pathogen as designated by WHO^57^. Attempts to identify phages against another colistin resistant *E. coli* U3790 was unsuccessful owing to the presence of the capsule as reported earlier and also possibly due to the intact prophages within its genome^58^. Prophages in bacterial genome can evolve mechanisms like blocking phage genome injection, blocking phage binding and preventing the interaction of phage receptor on the bacterial membrane to evade superinfection by other related phages, though the exact mechanism is still not known^59^. Nevertheless, bacteriophages targeting MDR *E. coli* U1007 were isolated from The Ganges River (designated as U1G), Cooum River (CR) and Hospital Waste water(M) (Figure S1). We recently reported a phage KpG, belonging to *Podoviridae*, specific to MDR *K. pneumoniae* from Ganges, which was able to curtail the host’s planktonic and biofilm mode of growth^33^. There are other numerous reports available on rich diversity of bacteriophages against various pathogens being isolated from The Ganges^60,61^. This is attributed to the origin of Ganges The Himalayan permafrost, which has trapped bacteriophages from a long period and is released gradually while melting and hence it forms a seed source of bacteriophage^62^. Both Cooum River and Hospital wastewater are likely to harbor a lot of MDR bacteria. Depending upon the origin, hospital waste water is likely to harbor MDR microbes from 0.58 to 40%^63^ As the prevalence of drug resistant microbes are likely to be higher in hospital wastewater, the propensity to harbor phages that target drug resistant microbes are also high. TEM imaging revealed that U1G belongs to the *Podoviridae* family, containing an icosahedral head and a short non-contractile tail. Whereas CR phage possessed a shorter head and a moderate tail and is likely to belong to *Myoviridae*. On the other hand M phage had a moderate sized head and a long tail (Fig. 1) hence it probably belongs to *Siphoviridae*. Thus in this study we had successfully isolated phages belonging to three different families yet targeting the same host. A recent study has shown that nettle manure harbored phages belonging to *Siphoviridae* and *Podoviridae* targeting the same plant pathogen *Pseudomonas syringae* pv. tomato^64^. However, genome analysis of U1G by PHASTER revealed the presence of RNA polymerase based on which, U1G can be designated under *Schitoviridae*. Recent reports show that only 115 members that were classified under this newly proposed family *Schitoviridae*^49^. Interestingly we also observed during whole genome BLAST analysis that a prophage in *Enterococcus faecium* strain ME3 chromosome displayed 95.94% identity (with 91% coverage and e-value of 0) with U1G phage genome but the presence and probable expression of antirepressor protein in U1G (Fig. 2) might favor its lytic life cycle. Presence of a highly homologous prophage genome in *Enterococci* imply that U1G might possibly use *Enterococcus faecium* as a host, we tried to infect a reference strain of *Enterococcus faecium* with U1G using spot test, our attempts to infect was unsuccessful which could possibly be attributed to genome harbored prophage in the reference *E. faecium* strain, which prevents super infection by a similar phage. One step growth data showed that all three phages had a short latent period but with varying burst size ranging from 114 PFU/cell to 150 PFU/cell (**Figure S5**). The exponential growth of phage and its lytic efficiency is majorly dependent on latent period and burst size. Larger burst size usually results from a long latent period and vice versa. However, a phage with shorter latent period and relatively good burst size possesses an enhanced capability to lyse host cells faster^65^. Experimental evidences and predictive modeling indicate that host cell densities and phage latent periods are inversely related and phages could be evolving towards a latent period optimum which tends to maximize the population of phages that grows in the presence of a specific quality and quantity of host cells^65^. Host specificity assay revealed that all three phages U1G, CR and M were highly specific to its host U1007 and all phages exhibited a faint lysis zone against U2354 strain (Figure S6). Interestingly, ANI matrix based phylogenetic genome analysis of three *E. coli* clinical isolates U1007, U2354 and U3790 show that they cluster together with > 97 percent genome similarity and exhibits good similarity with genomes of Enterroaggregative *E. coli* (Figure S7). Ability of all three phages from diverse sources to exhibit lytic ability against U1007 and a faint lytic potential againt U2354 imply that host genome similarity could also be used to predict the host range of phages. No clearance or appearance of plaques were seen for CR and M phages in other clinical isolates, including the reference strain MG1655. Whereas U1G exhibited a faint zone against another colistin resistant strain U3790, this led to a hypothesis that the phage U1G might exhibit preference towards colistin resistant *E. coli* although it also exhibited faint zone against colistin sensitive U2354 strain. As colistin resistance confers chemical alteration to LPS^66^, it is likely that altered LPS could serve as a receptor for phage entry, which was also predicted by ML algorithm (Table 2). Previous report showed that a panel of colistin resistant *K. pneumoniae* were more susceptible to lytic phage, isolated from sewage water, than to their respective colistin susceptible strains^67^. The isolated phages were observed to be negatively charged and since colistin resistant strains in general possess reduced cell surface negative charge, electrostatic interaction might have favored the enhanced susceptibility of colistin resistant strains to the phages^67^. As the host (U1007 strain) displayed colistin heteroresistance (Figure S10), it is likely to exhibit variable suseptiblity to colistin and could not be completely eliminated by colistin, in such a scenario, phage-antibiotic combination is likely to be more effective since the phage like U1G employs a different host receptor (ompC) for entry which unlike LPS O antigen is unaffected by colistin resistance. Thus the Phage antibiotic combination will take care of both colistin suseptible and colistin resistant subpopulations. Indeed our time kill data for triple phage combination **(**Fig. 5) reveals that phage colistin combination is able to cause a significant 2.5 log decline in cell counts relative to either phage treatment/colistin treatment alone against colistin heteroresistant *E.coli*. In the current study, we employed a machine learning-based approaches for host receptor prediction, specifically based on the RBP sequence. To the best of our knowledge, this is the first reported study on host receptor prediction utilizing this ML-based approach. Most studies primarily focus on predicting the hosts of bacteriophages rather than the receptors^44,46^; The advantage of using machine learning tools to predict host cell surface receptors is that it will reduce significant time and labor as conventional phage adsorption studies to identify host receptor will involve laborious screens with large mutant libraries and for clinical isolates such mutant libraries needs to be created first which is cumbersome^68^. Machine learning tools will considerably reduce the labor by pinning down on a handful of putative receptors for which knock outs (even in clinical isolates) can potentially be developed and screened. Random forest algorithm using all sequence features and features based on ANOVA approach identified LPS O antigen as the host receptor employed by the phage tail protein for entry into the host. As LPS O antigen knock outs are difficult to generate in an isogenic background, we attempted to physiologically validate other receptor(s) that are predicted by the feature selection approach. Random forest algorithm upon L2 regularization identified OmpC as one of the host cell surface receptors for U1G phage. As OmpC expression is modulated by medium osmolality^53,54^, to validate the role of OmpC as a host cell surface receptor, U1G phage was exposed to cells grown under conditions that either upregulate OmpC (high osmolality) or downregulate OmpC (low osmolality) and equal ratio of phages to bacterial cells were maintained for both treatments and the results showed that phage titers were indeed high (Fig. 4A) when OmpC is upregulated (Fig. 4B), this validates the prediction by RF algorithm (Table 2) that OmpC is the host cell surface receptor for the U1G phage. Future studies will attempt to create knock outs of predicted receptors (LPS O antigen, LamB, FhuA, OmpC, OmpF, TonB) in isogenic (U1007) background and reaffirm predictions of host cell surface receptors made by ML algorithms for all the three phages. The current ML algorithm takes into account only the nucleotide and protein sequence features of RBPs. However, further enhancements can be made by incorporating structural features once the relevant data becomes available^69^. Relative to CR and M phages, U1G phage was more thermostable and it retained 50% viability at 65 °C (Figure S8) . Conversely M and CR phage displayed greater viability in alkaline pH relative to U1G phage (Figure S9). Interestingly, pH is also known to affect the expression of OmpC^70^ . A previous report has shown that acidic pH stimulates OmpC whereas alkaline pH stimulates OmpF^69.^ Since OmpC is the receptor employed by U1G, reduced phage titers at alkaline pH for U1G might as well be attributed to reduced expression of OmpC at alkaline pH.

In vitro time kill study with Phage combinations, individually and in combination with colistin showed that phage combination along with colistin showed better ability in restricting regrowth relative to treatment with monophages (Fig. 5). Usually one would expect a drastic reduction when using phage combinations, as all three phages belonged to different families as evident from the morphology (Fig. 1). But phylogenetic analysis based on genome similarity showed that U1G is distinct from CR and M which are quite closely related to each other (Fig. 3). Earlier studies have shown that co-infection by different phages on the same host might result in smaller burst size and infection exclusion^71^, which might account for the relatively lower titers observed in triple phage combinations. LPS O antigen is predicted as one of the targets for the phages by ML tools (Table 2), alteration of O antigen is a common phenomenon so bacteria gaining resistance to monophages is possible which necessitates the use of phage cocktails. It is likely that the bacteria during its attempt to develop phage resistance might partially lose resistance to colistin and hence the combination can achieve enhanced killing than when individually treated. As reported in many earlier studies, bacterial resistance (regrowth) was observed after 2 h of phage treatment (Fig. 5). Despite regrowth, phages cocktail in combination with colistin was able to restrict the bacterial regrowth significantly, which plateued around 6 h (Fig. 5). Ability to restrict phage growth especially by cocktails signify that the phages in the cocktail target different receptors and possibly co evolve thereby restricting bacterial regrowth by 6 h. Previous studies have reported that evolution of resistance to phages incurs a fitness cost for the bacterium^72^. A recent study has shown that resistance to phages HP3 and ES17 targeting UPEC resulted in different mutant strains with fitness costs that range from defects in LPS biosynthesis, poor growth in pooled urine, reduced adherence and increased suseptiblity to membrane perturbing antimicrobials and inability to colonize well in murine UTI infection model^73^. Interestingly in some instances phage-bacterial co evolution allows both of them to survive without eliminating each other as seen in lambda phage wherein, mutation of LamB receptor by the host resulted in evolution of phages with high affinity of LamB initially and later there was a switch in the receptor from LamB to OmpF through amino acid substitutions in the tail fibre J protein^74^. Co evolution studies signifies that evolution of phages in response to bacterial resistance can retain the phage efficiency and curtail the bacterial growth during phage therapy. Zebrafish model has been used to study the efficiency of bacteriophages in curtailing infections caused by *P. aeruginosa, K. pneumoniae, E. coli* and *E. faecalis*^33,75–77^ . Nevertheless, the majority of studies have compared the effect of antibiotics and phage therapy and very few reports have studied the combination of antibiotic and phage therapy. In our earlier study, we found that the combination of Streptomycin and KpG (*Podoviridae* phage specific to *K. pneumoniae*) curtailed the infection by 98% relative to untreated control, whereas KpG alone caused 77% reduction and only streptomycin resulted in 63% reduction in colony counts^33^. In the present study, a drastic decline in bacterial bioburden of upto 2.2 log CFU was observed when bi-phage (U + M) combination was used along with colistin relative to bi-phage treatment alone in fish infection study (Fig. 6). A similar trend was not observed when all three phages were used in combination with colistin, which resulted in modest 0.5 log CFU difference between phage cocktails with and without colistin (Fig. 6).Enhanced phage activity in the presence of subinhibitory concentrations of antibiotic termed as phage antibiotic synergy (PAS) as reported by Comeau et al., 2007^78^ was observed by many others as reviewed by North et al.,2019^38^ and is attributed to enhanced burst size or collateral sensitivity to antibiotics due to phage resistance^79^. Future studies of one step growth curve with sub MIC levels of colistin can unravel whether enhanced burst size triggered by the antibiotic is responsible for PAS observed in the present study. Phage antibiotic combination treatments can reduce rate of resistance evolution to either phage or antibiotic or for both^80^ . In the present study, U1G + CR + M & U1G + M along with colistin caused a significant 3.0 and 3.5 log decline in bacterial CFU respectively (Fig. 6), which reaffirms the ability of phage cocktail to restrict bacterial bioburden in vivo and can be potentially evaluated for its efficacy in mammalian models.

## Materials and methods

### Screening for bacteriophages

Bacteriophages were screened against the MDR *E. coli* strains U3790 and U1007 strains from different sources. The Ganges river, The Cauvery river, Cooum River, Hospital waste water, pond water samples, samples from cowsheds and soil samples from farmland were screened for phages using spot test^81^. Briefly, the samples were incubated with host culture at 37 °C and after incubation for 16–24 h, the samples were centrifuged at 5000 rpm for 15 min. 3–5 µl of the supernatant harboring phages were spotted on agar plates overlaid with the host (clinical isolates of *E. coli* U3790/U1007) and the plates were incubated for 18–24 h at 37 °C. Presence of bacteriophages against a specific host strain can be identified by appearance of clear zones or plaques on the agar plates.

### Genome sequencing and analysis of U1007 strain

Genome sequencing of U1007 was performed essentially as reported earlier (Sundaramoorthy et al.^58^). The whole genome sequencing library was prepared using QIAseq FX DNA Library Kit for Illumina. The DNA was amplified by 4 cycles of PCR with the addition of HiFi PCR master mix and Primer mix and the amplified products were then purified using 1 X AMPure XP beads and the final DNA library was eluted in 15 µl of 0.1X TE buffer. The library concentration was determined in a Qubit.3 Fluorometer. The library quality assessment was done using Agilent D5000 ScreenTape System in a 4150 TapeStation System. The sequencing of the *E. coli* clinical isolates was done using Illumina HiSeq 2500 in high throughput run mode using 2 × 125 bp format. The sequencing library was prepared using TrueSeq DNA library sample prep kit v2 following the manufacturer’s guidelines. The Read quality of the Sequenced Clinical Isolates was verified using FastQC (version 0.11.9) to identify presence of adapter content. The adapter sequences were removed and the quality of the reads were verified using TrimGalore (Version 0.6.4) to ensure adapter sequence free reads for further processing. Paired end read pairs were then obtained prior to assembly using fastq-pair (v1.0). The QC passed paired read pairs were then assembled de novo using SPAdes Aligner (Version 3.13.0). Quast (Version 5.0.2) was used to assess the quality of the assembled genomes. Genome Annotation and functional categorization were performed using the RAST server (Version 2.0). The AMR genes present in the clinical isolate of *E. coli* were identified by Resfinder, RAST and Roary.

### Isolation and purification of bacteriophages

In order to isolate the phage, the phage containing supernatant was filtered through 0.45 µm and 0.22 µm syringe filters^82^. The filtered phage lysate was serial diluted in SM buffer (100 mM Sodium Chloride, 8 mM Magnesium sulphate, 50 mM Tris hydochloride (pH 7.5)) and each dilution was allowed to incubate with mid log cells of the host. After 20 min of incubation, the phage–host mixture was added to 5 ml of soft agar (0.7% Luria Bertani Agar) and overlaid on Nutrient Agar plates. The plates were allowed to solidify, and were incubated at 37 °C for 18–24 h and observed for plaques. A single plaque was then picked and resuspended in 1 ml of SM buffer, serial diluted, mixed with host cells and overlaid on nutrient agar plates as mentioned earlier. The procedure was repeated three times to obtain triple purified plaques containing identical morphology. The phage titer was determined at each step and represented as PFU/ml.

### TEM imaging

The triple purified phages were enriched using the specific host (*E. coli* U1007 strain) to obtain a high phage titre (> 10^12^ PFU/ml). 10 µl of the high titer phage lysate was added to the carbon coated copper grid and was allowed to attach for 2 min^83^. The excess phage lysate was immediately removed carefully using a filter paper and then the phages were stained with 2% uranyl acetate for less than a minute. The excess stain was removed, the grid was allowed to dry and was then observed under a FEI Tecnai G^2^ 20 S-Twin Transmission Electron Microscope (TEM) at 200 kV. The TEM images were analysed for the phage morphology to discern the family to which phage belongs.

### One step growth curve

Burst size and latent period was determined for the isolated phage using one-step growth curve analysis^84^. Mid log cells of the host bacteria were mixed at a multiplicity of infection (MOI) of 1 and incubated at 37 °C for 5–10 min for adsorption. The cells with adsorbed phages were harvested by centrifugation at 5000 rpm for 10 min and resuspended in Nutrient Broth and incubated at 37 °C. Phage titer was determined for the samples at different time intervals until 60 min. Latent period is the time interval between the phage adsorption to the host and the host cell lysis. Burst size is the number of phages from an infected host cell and is calculated as the ratio of average PFU/ml of latent period to average PFU/ml of last three time points. The experiment was performed in triplicates and was reported as SD from the mean.

### Host specificity

The host range of the isolated phage was determined by spot assay with 10 microbes which included 8 different *E.coli* strains, *Salmonella enterica* serovar Typhimurium and *Klebsiella pneumoniae*^85^. The bacteria to be tested as host were independently grown in LB broth till it attained mid log phase (0.4 OD at 600 nm). 300 µl of the midlog phase bacterial culture to be tested as host was added to soft agar, overlaid on nutrient agar plates and the top agar was allowed to solidify. Post solidification, 10 µl of triple purified phage lysate was spotted on overlaid plates and were allowed to dry. The plates were incubated at 37 °C for 18 h and then observed for clear zones. Presence of plaques represent the susceptibility of the bacterial culture to the purified phage.

### Temperature and pH stability

The temperature sensitivity of the phage was studied by incubating phage at different temperatures from 4 °C, 16 °C, 25 °C, 37 °C, 45 °C, 65 °C and 95 °C for 1 h^86^. Post incubation, phage were made to interact with the host and and the phage–host mixture was added to 5 ml of soft agar (0.7% Luria Bertani Agar) and overlaid on Nutrient Agar plates. The plates were allowed to solidify, and were incubated at 37 °C for 18–24 h subsequently, plaque titers were determined. Similarly, pH sensitivity of the phage was analysed by incubating the phage at different pH 3.0, 5.0, 7.0, 9.0 and 11.0 for 1 h. After incubation, the phage lysates were made to adsorb with the host and plated by agar overlay method as mentioned above to determine phage titers. After 18–24 h of incubation, plaque titers were determined and were expressed as PFU/ml^34^. All experiments were performed in triplicates and represented as the percent survival rate.

### In vitro time kill study

In order to evaluate the efficiency of phage to inhibit the growth of antibiotic resistant strains in vitro, time kill study^87^ was performed with monophages, and different phage cocktails both with and without colistin. Mid log cells were subjected to different treatments with phages alone and phages in combination with colistin and at regular time intervals viz., 0, 2, 4, 6 and 24 h, the samples were withdrawn, serial diluted and plated on LA plates. After overnight incubation at 37 °C, the CFU/ml was calculated and the difference in colony counts due to phage treatment was analyzed.

### In vivo toxicity study

The toxic effect of phage on zebrafish (*Danio rerio*) PET strains^88^ was discerned by injecting 10 µlof purified phage lysate (10^4^, 10^10^, and 10^12^ PFU/ml) intramuscularly control fish were injected with 10 µl of sterile PBS. Phage injected fish and uninjected control fish were monitored for 48 h and then the fish were euthanised and dissected. The brain and liver tissue were isolated, homogenized and the clear supernatant was used for further analysis. Brain and liver enzyme profiles were evaluated using acetylcholine iodide and α/β naphthyl acetate as substrates, respectively^89^. Any significant changes in enzyme levels relative to untreated control was deemed to be toxic to zebrafish.

### In vivo infection

The CPCSEA guidelines for laboratory animal facilities (Central Act 26 of 1982) were adhered in all in vivo experiments. The protocols were approved by Institutional Animal Ethics Committee (CPCSEA-493/SASTRA/IAEC/RPP) of SASTRA deemed University, India and experiments were performed by following protocols approved by Institutional Animal Ethics Committee, SASTRA deemed University, India. Briefly Adult zebrafish (*Danio rerio*) irrespective of sex, measuring 4 to 5 cm in length, weighing approx.300 mg, were purchased from a local aquarium. Animal acclimatization was performed following established protocols. The efficacy of monophage and phage cocktail alone or in combination with colistin in preventing growth of MDR strain was evaluated using zebrafish infection^90^. 10 µl of *E. coli* U1007 (~ 10^8^ CFU/ml) was injected intramuscularly and 2 h post infection the fish were split to receive different treatment combinations viz., monophage (U or M or C)/phage cocktail (UM, MC, MCU), monophage/phage cocktail along with colistin and colistin alone. 24 h post treatment, the fish from different treatment groups were euthanised via Immobilization by submersion in ice cold water, the infected muscle tissue was dissected, homogenized, serially diluted in sterile PBS, and plated on LA plates. After incubation for 24–48 h at 37 °C, colony counts were determined and represented as mean CFU from triplicate values.

### Genome sequence and analysis of bacteriophages

The phage DNA was extracted using Cetyl Trimethyl Ammonium Bromide (CTAB) DNA precipitation method^91^. 1.5 ml of purified high titer phage lysate was incubated at 22 °C for 15 min with 10 ng of RNase A and 10 U of DNase I. Post incubation, 80 µl of 0.5 M EDTA and 50 µg of Proteinase K was added and was maintained at 45 °C for 15 min, followed by addition of 5% CTAB and incubation in ice for 15 min. The precipitated DNA was harvested at 8000 g and the pellet was resuspended in 1.2 M NaCl. The DNA was further precipitated and washed with ethanol. The pellet was air dried and suspended in 10 mM Tris buffer, which was stored at -20 °C until use. The DNA was quantified using Qubit Fluorometer and was then sequenced using Oxford MinIon Nanopore sequencer. Library preparation was performed as per manufacturer’s instruction using Ligation sequencing kit (SQK-LSK109) and the sequencing run was performed without live basecalling. Basecalling and demultiplexing were performed using Guppy and de novo assembly of the reads were done using Canu Assembly software for the U1G genome^92^. For the CR and M phage genomes, basecalling and demultiplexing of the Minion raw data were done using Guppy. Adapters were removed using Porechop (https://github.com/rrwick/Porechop) and further processing using fastp^93^ and only reads above 200 bp were retained. De novo assembly of the reads was done using Flye (https://github.com/fenderglass/Flye) with asm_coverage of 50 and four polishing iterations using an expected genome size of 60 Kb. The genome assembly and completeness were assessed using QUAST Version 5.2.0(https://github.com/ablab/quast). The assembled reads were annotated using Rapid Annotation using Subsystem Technology (RAST)^94^. The assembled genome was searched against nucleotide database available at National Center for Biotechnology Information (NCBI) using Basic local alignment search tool (BLASTn) to identify close homologs and the top phage hits with query coverage above 80 were considered for the analysis^95^. Presence or absence of tRNAs were identified using tRNAscan-SE search server^96^ and the reads were also fed to PHASTER to determine intactness^97^. The Average Nucleotide Identity (ANI) between the close homologs of U1G was calculated using the ANI/AAI-Matrix tool (http://enve-omics.ce.gatech.edu/g-matrix/)^98^. Phylogenetic tree of phoage homologs was obtained using ANI-Distance clustering method, UPGMA. For RBP homologs, tree was constructed using the Maximum Likelihood method. The tree was visualised using iTOL v6 (https://itol.embl.de/)^99^. Antibiotic resistant and virulent genes in the phage genome were analysed using ResFinder and Virulence Finder of CGE^100,101^. Potential RBP sequences in the annotated genomes of all three phages were identified by conducting a BlastP search of the amino acid sequences of the assembled bacteriophage genomes against the RBP protein sequence database locally^46^.

### *E. coli* bacteriophage host receptor prediction using machine learning based multi class classifier

The initial entries of the RBP nucleotide and protein sequences were obtained from the database curated by a study^46^ conducted to predict bacterial hosts which consisted of 1232 RBP sequences belonging to nine different bacterial hosts, out of which 400 had *E.coli* as the bacterial host. The bacteriophages that targets *E.coli* as the host were mapped to the PhRED database^102^ which provides information on the corresponding receptor proteins involved in the bacteriophage host interaction of which only 71 RBP entries had the receptor information. For RBP database entries with PhRED receptor information, the sequence information was updated by a manual search of annotated proteins and CDS from the genome sequence of bacteriophages. Common search terms used were ‘tail fiber protein’, ’tail protein’, ‘tail fiber’ along with the bacteriophage name. RBP database entries with sequence information pertaining to the receptor information were updated from a literature survey of experimental studies conducted on bacterial host receptor interactions^103,104^. The curated database of RBP and receptor information with 160 entries were used for building the ML model (Supplementary file 2). The RBP sequences were presented as a vector of numerical features extracted from both the nucleotide and protein sequences based on the script available with the study^46^. A total vector of 218 numerical features (Supplementary file 2) was retrieved for each of the RBP dataset entries.

Data preprocessing involves checking for null values in the dataset, obtaining a balanced dataset, and training it. The numerical features obtained from the nucleotide and protein sequences were normalized using MinMaxScaler from the Scikit-learn package(v 1.0.2)^105^ to the range of 0 to 1. Upon performing exploratory data analysis, it was found that the RBP dataset is imbalanced in nature in terms of the target variable distribution with some labels having just a single entry. In order to address this issue, only RBP entries with label count two or more were included and RBP entries with single-entry outliers were removed, setting the final size of curated dataset to 155. We included SMOTE (Synthetic Minority Oversampling Technique)^106^, a method of oversampling that creates artificial samples from the class with the lowest count. For training the classifier, SMOTE is utilized to create a training set that artificially balances the class distribution. Three classification algorithms, Random Forest, Multinomial Logistic Regression and Decision Tree were used for the construction of the multiclass classification models and were validated using the Nested Cross Validation approach. The range of parameters to be run for the classification models was defined. Multinomial Logistic Regression: C: [0.001, 0.01,0.1,1,10,100,1000]andDecisiontreeClassifier: max_features":[2, 4, 6],"criterion":["gini","entropy"],RandomForestClassifier: n_estimators":[10, 50, 100], max_features: [5, 10, 15], criterion:["gini","entropy"] in order to run the Nested Cross Validation with an Outer Loop Fold of 10 and Inner Loop Fold of 5. In this study, we implemented two feature selection methods, Analysis of Variance (ANOVA) and L2 Regularization, and compared the performance of the trained model with those two datasets to that of the 218 Features incorporated dataset.

The performance evaluation scores like accuracy, precision, F-1 Score and Matthew’s Correlation coefficient (MCC) were calculated from the three categories of datasets namely with all features selected dataset with 218 features and ANOVA feature selected dataset with 30 highest scoring features, and L2 -Regularization selected Dataset with 110 features trained with the three classification algorithms. The ROC Curves were generated for each Dataset—Classification algorithm combination. The implementation of Classification algorithms and cross validation was done using the Scikit-learn package (version 1.0.2) available in Python^105^. After optimizing the ML model, the RBP sequences of the phages were used to predict the potential host receptors of the bacteriophages.

### Physiological validation of host receptor

To aid in validation of OmpC as predicted by RF algorithm, *E. coli* U1007 cells were inoculated from overnight grown culture in LB-NaCl containing either 0.1% sucrose or 10% sucrose and the cells were grown till they attained a cell density of 0.4 OD. Subsequently 300 µl of 0.4 OD cells were mixed with top agar and overlaid on bottom agar following solidification, different dilutions of phages were  spotted on bacterial lawn containing top agar. Following incubation at 37 °C for 6 h, plaques formed with cells grown under 0.1% sucrose was compared with plaques obtained from cells propagated in medium containing 10% sucrose.

### Gene expression profiling

*E. coli* U1007 cells were grown in M9 medium with either 0% sucrose or 10% sucrose. The cells were allowed to attain an OD of 0.6–0.7, following which cells were pelleted, washed and RNA was extracted from these mid log cells using AURUM Total RNA mini kit (Biorad, Hercules, CA). The isolated RNA was quantified using Qubit RNA HS Assay kit (Thermo Fisher Scientific, Waltham, MA) and equal amount of RNA was taken for conversion to cDNA using M-MuLV reverse transcriptase from RevertAid First Strand cDNA Synthesis Kit (Thermo Fisher Scientific, Waltham, MA). qRT PCR was performed using SYBR green based assay with QuantiFast SYBR Green PCR Kit (Qiagen, Hilden, Germany) using gene specific primers for OmpC and 16srDNA (internal control). RT-PCR run was performed using QuantStudio 5 thermal cycler [Applied biosystems, Waltham, MA) Following the run, melt curve analysis was performed to confirm amplification of only OmpC and internal control (16srDNA). Difference in gene expression between low osmolality and high osmolality groups were quantified using ∆∆ct method (Livak and Schmittgen)^107^.

### Statistical analysis

Statistical test for the groups were performed using graph pad prism software.[Graphpad Prism 8].

### Informed consent

Study is reported in accordance with ARRIVE guidelines.

## Conclusions

Our study revealed that the phage cocktails and colistin combination is effective in curtailing the growth of colistin resistant *E. coli* (U1007) both in vitro and in vivo*.* Machine learning tools predicted potential host cell receptors, among which, OmpC was validated as the host cell surface receptor for U1G by growth under different physiological conditions followed by the estimation of phage titers. As the lytic potential of U1G phage is enhanced by M phage in combination with colistin, Phage cocktail (U1G + M) and colistin has the potential to curtail colistin resistant *E. coli* in mammalian models.

### Supplementary Information

Supplementary Information 1.

Supplementary Information 2.

## Data Availability

The genome datasets generated and/or analysed during the current study are available in the GenBank repository, [*E. coli* U1007 Genome sequence (Accession No: PRJNA988283), U1G Phage (Accession No: MZ394712; Escherichia phage U1G, complete genome—Nucleotide—NCBI (nih.gov)) CR Phage (Accession No. OR061068; Escherichia phage CR01, complete genome—Nucleotide—NCBI (nih.gov) and M phage (Accession No. OR061069; Escherichia phage M01, partial genome—Nucleotide—NCBI (nih.gov)). All other essential data has been provided either in the main text or as supplementary information. Raw data can be shared upon request to sai@scbt.sastra.edu or sumamohan@scbt.sastra.edu.
