# Supplementary material for: Genome analysis of triple phages that curtails MDR E. coli with ML based host receptor prediction and its evaluation
Source: Sci Rep. 2023 Dec 27;13:23040. doi: 10.1038/s41598-023-49880-x (PMC10754912; doi:10.1038/s41598-023-49880-x)
Supplement: Supplementary file 1 — Supplementary Information 1. [file 41598_2023_49880_MOESM1_ESM.pdf]

Supplementary Information:

Genome Analysis of Triple Phages that curtails MDR *E. coli* with ML based host receptor prediction and its evaluation.

Vineetha KU[a$], Niranjana Sri Sundaramoorthy[a,b$], , Veena Nair, Kavi Bharathi, Jean Sophy Roy[a], Malarvizhi R, Sneha Srinath[c], Santhosh Kumar S[c], Prakash Sankaran, Suma Mohan S*[c] and Saisubramanian Nagarajan[a,d]*

[a] Center for Research on Infectious Diseases (CRID), School of Chemical and Biotechnology, SASTRA Deemed University, Tamil Nadu, India

[b] Translational Health Sciences Technology Institute, Faridabad

[c] Department of Bioinformatics, School of Chemical and Biotechnology, SASTRA Deemed University, Tamil Nadu, India

[d] Antimicrobial Resistance Lab, ASK-I-312, School of Chemical and Biotechnology, SASTRA Deemed University, Tamil Nadu, India

$- Equal contribution by both the authors

*Jointly Communicated

Communicating Authors

Dr. Saisubramanian Nagarajan

Mail id: sai@scbt.sastra.edu

Dr. Suma Mohan S

Mail id: sumamohan@scbt.sastra.edu

Figure S1: Spot assay showing the presence of bacteriophages specific to U1007. 1- Water sample from Madurai Pond 1, 2- Cow's urine sample from Madurai, 3- Water sample from Madurai pond 2, 4 – Urine sample from cow shed at Perambalur, 5 – Dung sample from cow shed at Perambalur, 6 – Water sample from Cauvery river, 7 – Urine sample from SASTRA cow shed, 8 – Dung sample from SASTRA cow shed, 9 – Soil sample from a corn farm, 10 – Soil sample from a onion farm, 11 – Water sample from Ganges river 12- Hospital sewage sample, 13- cuvam river, 14- sewage sample from Taramani area, 15- Railway station area.

A) U3790

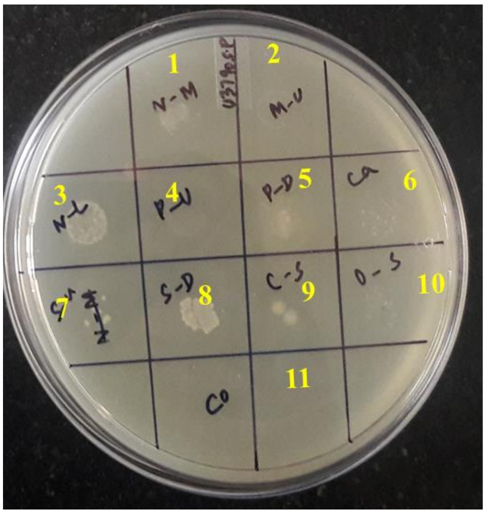

B) U1007

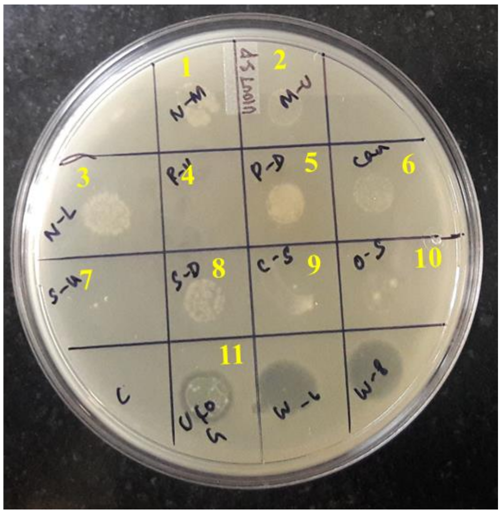

B) U1007

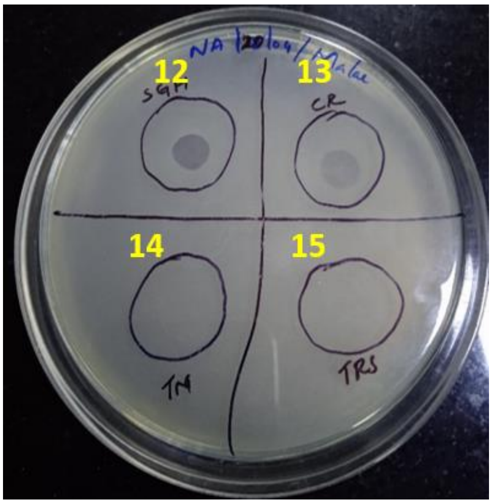

Figure S2: Genome alignment of the close homologs of Escherichia phage (A)U1G, (B) CR and M with Mauve.

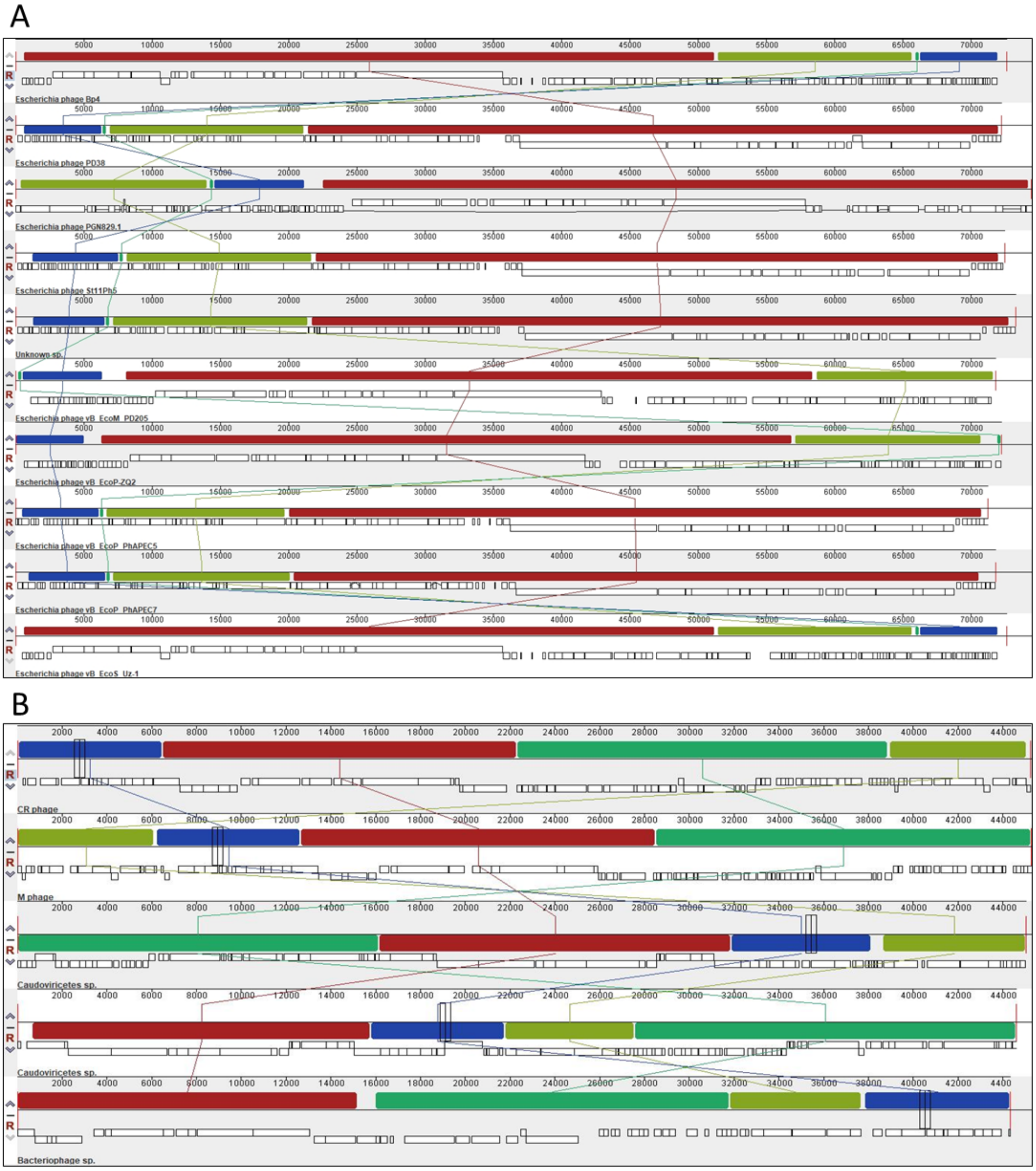

Figure S3: Phylogenetic tree of the potential RBP proteins identified from (A) U1G, (B) CR and (C) M phage. Phylogenetic tree of the RBP protein constructed with the Maximum Likelihood method with 500 bootstrap values using MEGA11. The homologs of the RBP proteins of U1G, CR and M phages from other bacteriophages were obtained using BLASTp search.

A

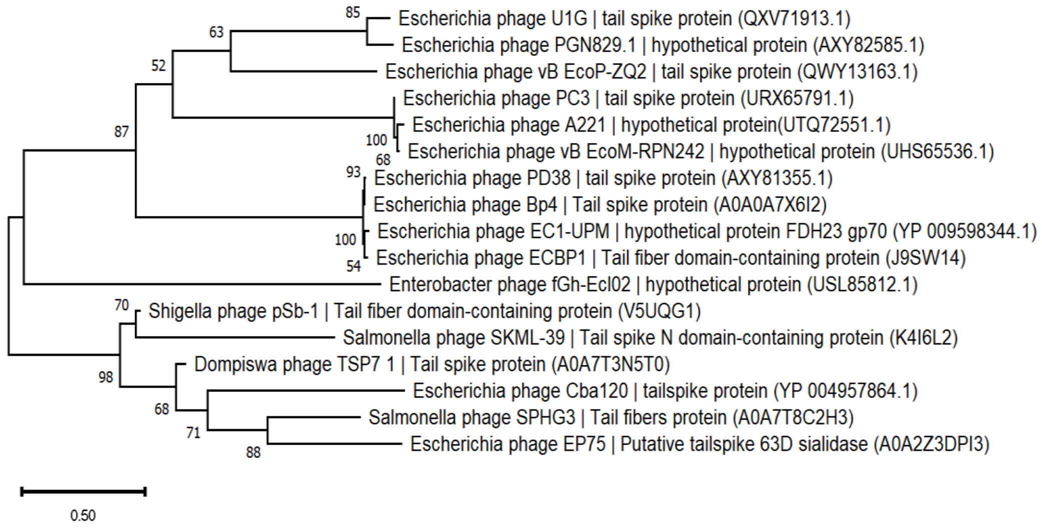

B

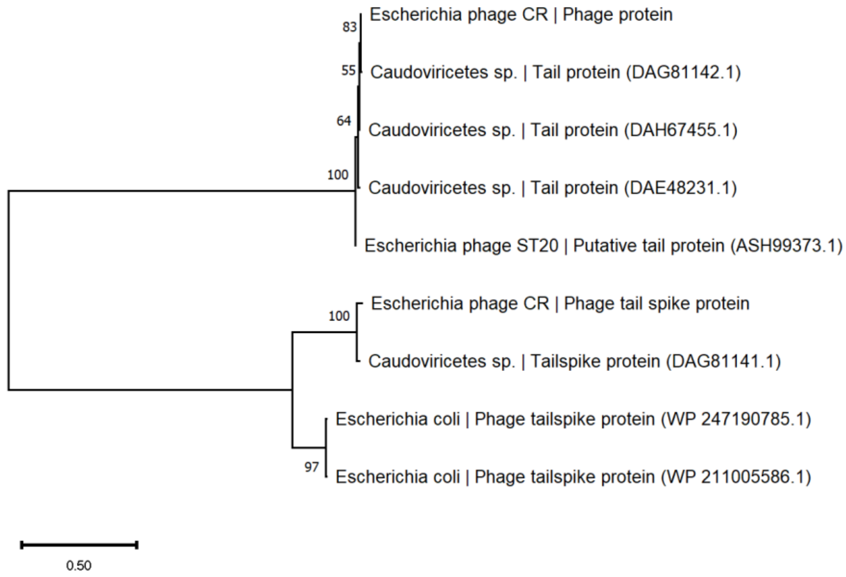

C

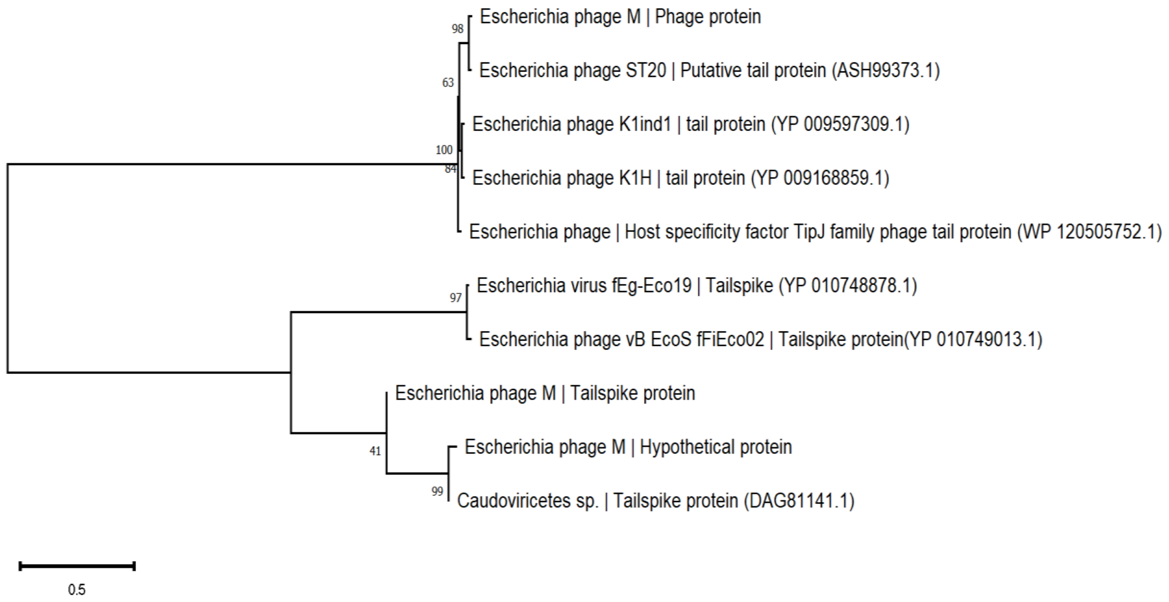

Escherichia phage M | Phage protein
Escherichia phage ST20 | Putative tail protein (ASH99373.1)
Escherichia phage K1ind1 | tail protein (YP 009597309.1)
Escherichia phage K1H | tail protein (YP 009168859.1)
Escherichia phage | Host specificity factor TipJ family phage tail protein (WP 120505752.1)
Escherichia virus fEg-Eco19 | Tailspike (YP 010748878.1)
Escherichia phage vB EcoS fFiEco02 | Tailspike protein(YP 010749013.1)
Escherichia phage M | Tailspike protein
Escherichia phage M | Hypothetical protein
Caudoviricetes sp. | Tailspike protein (DAG81141.1)
98
63
100
84
97
41
99
0.5

Figure S4: The receptor label distribution in the dataset with multiple entries.

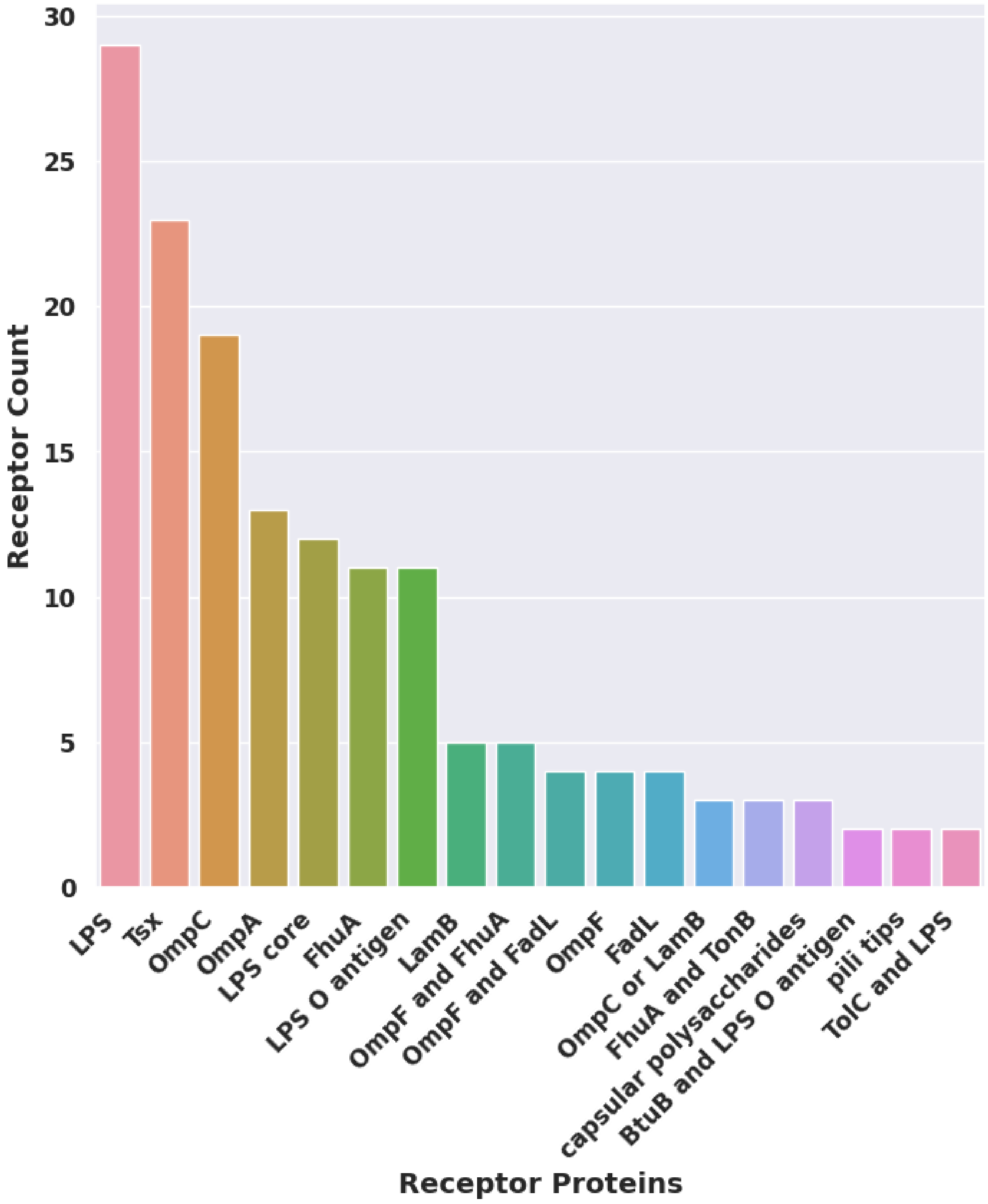

Figure S5: One-step growth curve of U1G, CR and M phages

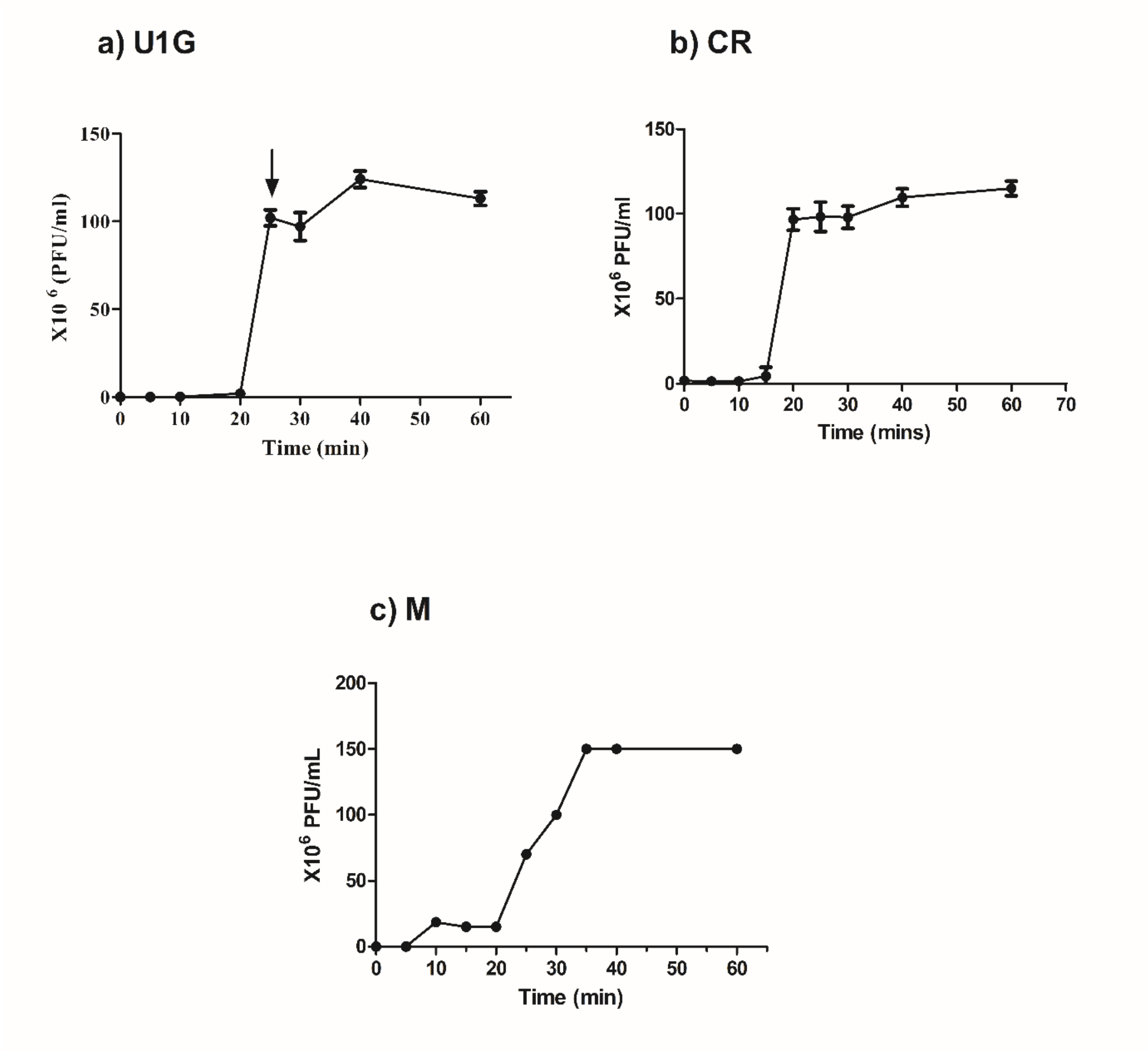

Supplementary Figure S6a: U1G is highly specific to U1007, with a slight lysis against U3790 and U2354.

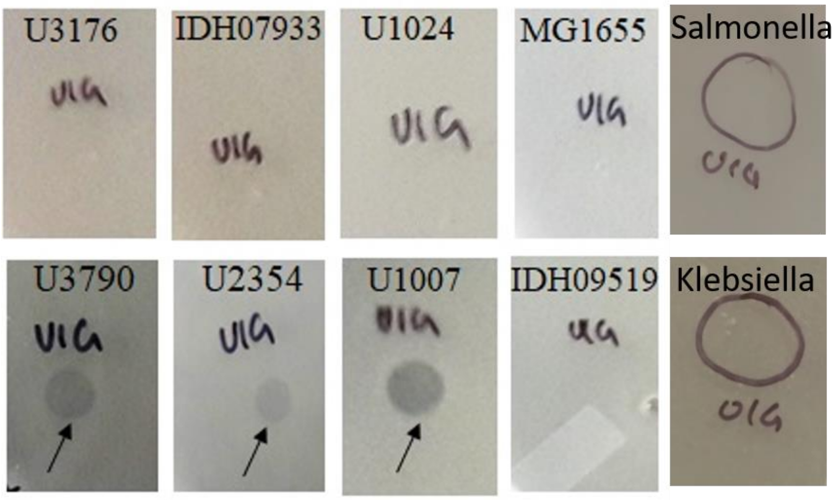

Figure S6b: M is highly specific to U1007, with a very faint lysis against U2354.

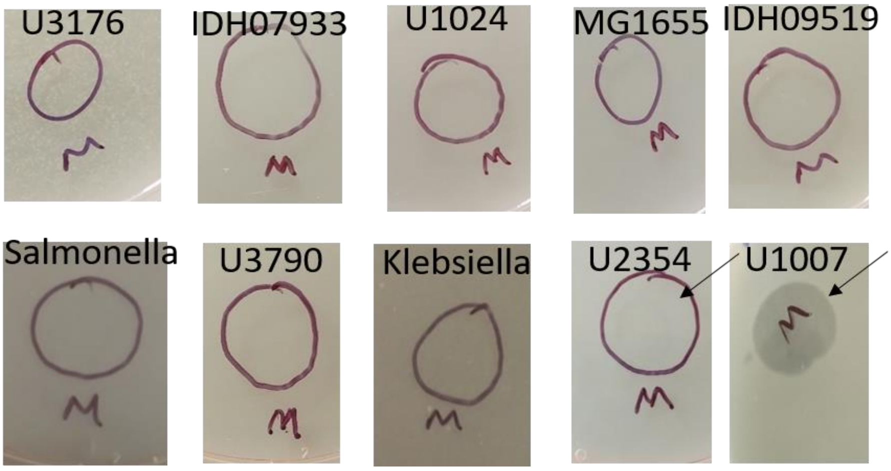

Figure S6c. CR is highly specific to U1007, with mild lysis against U2354.

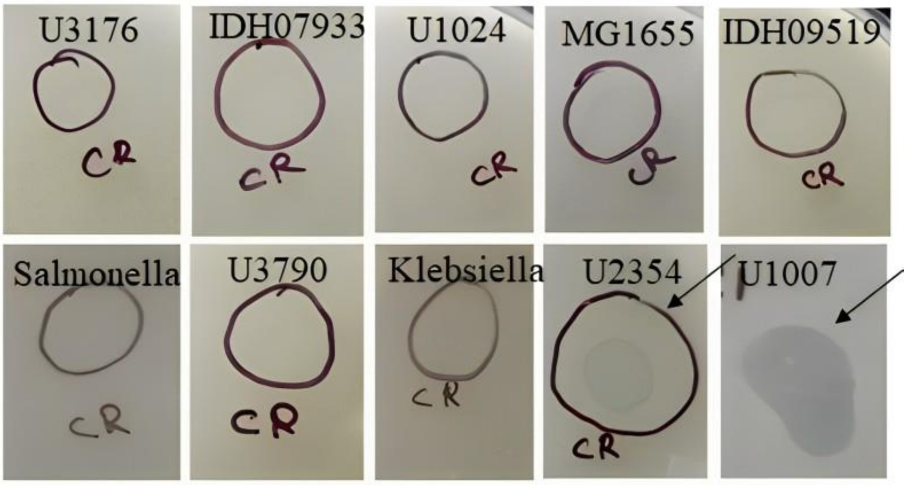

Figure S7: Genome similarity can aid in host specificity prediction. Phylogenetic tree of Clinical isolates of *E.coli* constructed using ANI Matrix depicting genome relatedness of U1007, U3790 and U2354.

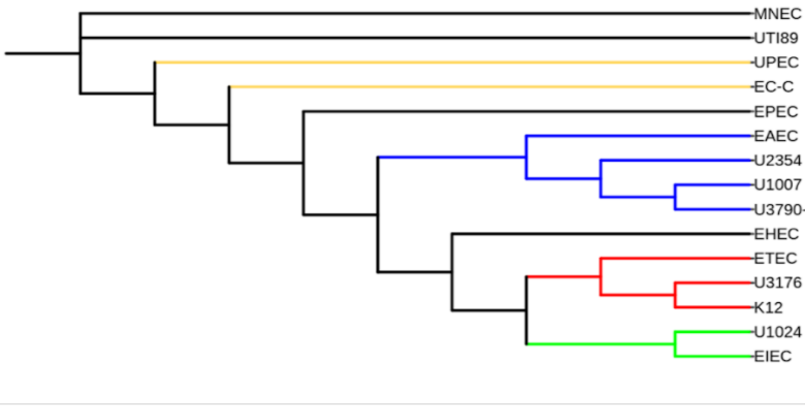

Figure S8: Representative Temperature stability data of U1G, CR and M phages

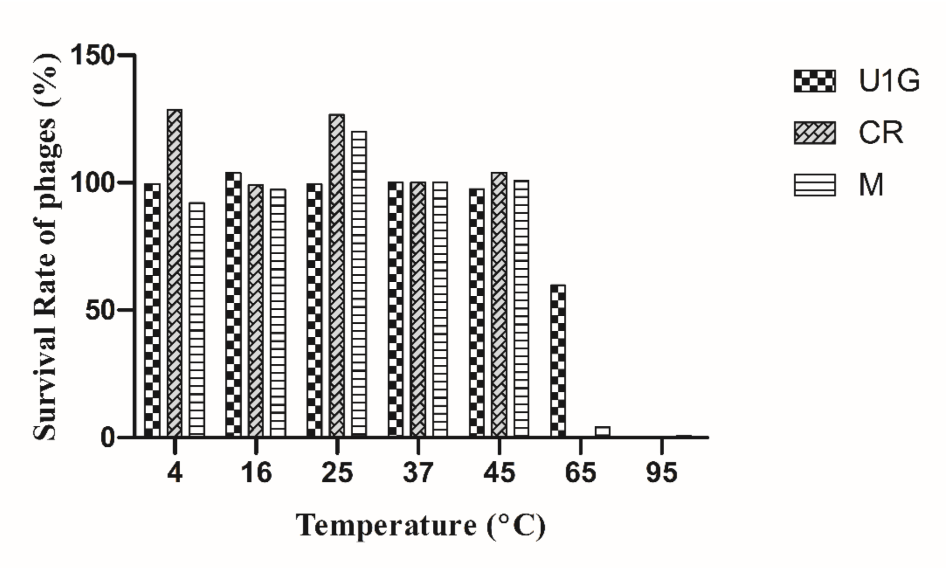

Figure S9: Representative pH stability data of U1G, CR and M phages

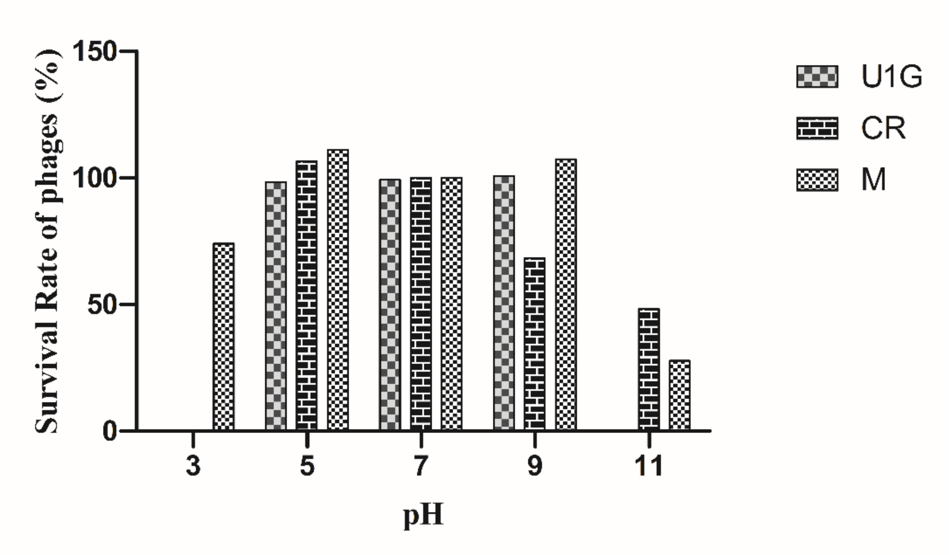

Figure S10: *E. coli* Strain U1007 is heteroresistant to colistin

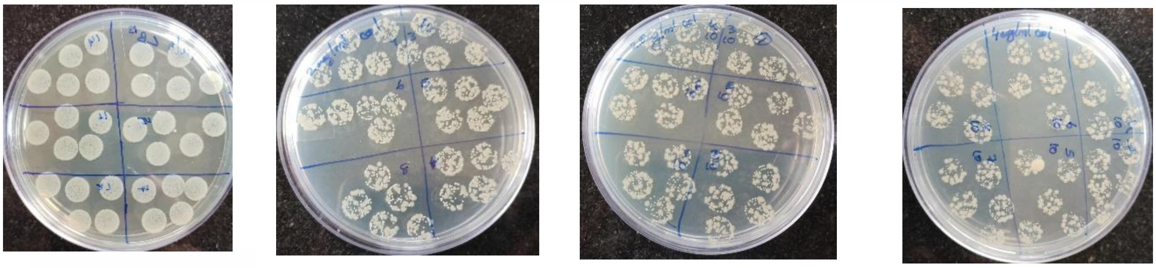

Colistin :0 µg/ml 2 µg/ml 2.5 µg/ml 4 µg/ml

Heteroresistance Calculation

$$\frac{\text{No.of colonies on colistin plate * Dilution factor}}{\text{No. of colonies on antibiotic free plate * Dilution factor}}$$

$= 30*\ 10^{8}/\ 609*10^{10} = 0.000492$

Figure S11: Representative toxicity testing of phages on zebrafish. Phages were injected in muscle tissue and 48h post injection, liver and brain were dissected

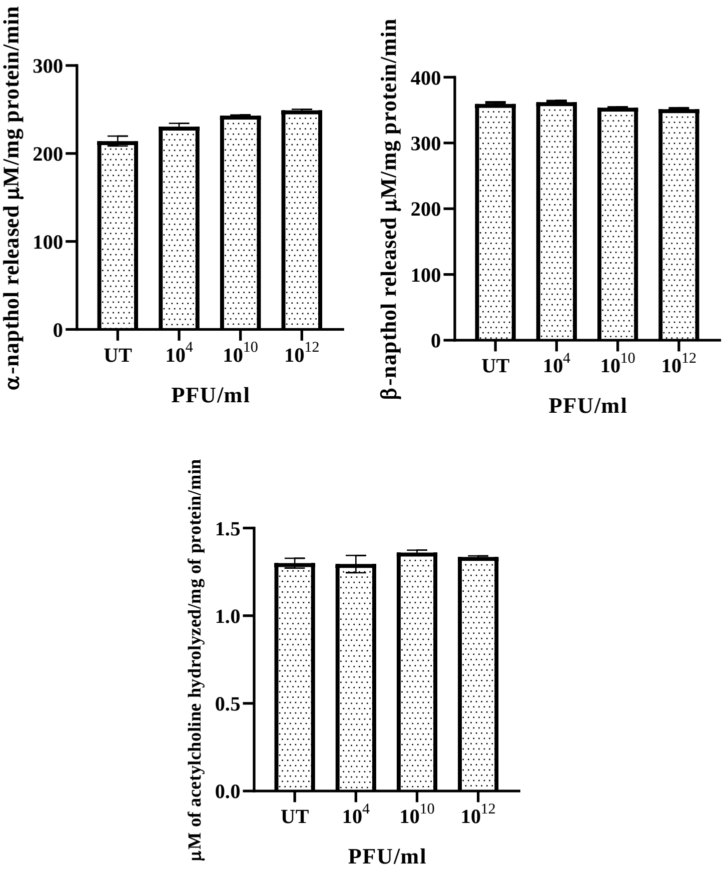

Figure S12: ***In vivo*** **infection study with monophages**

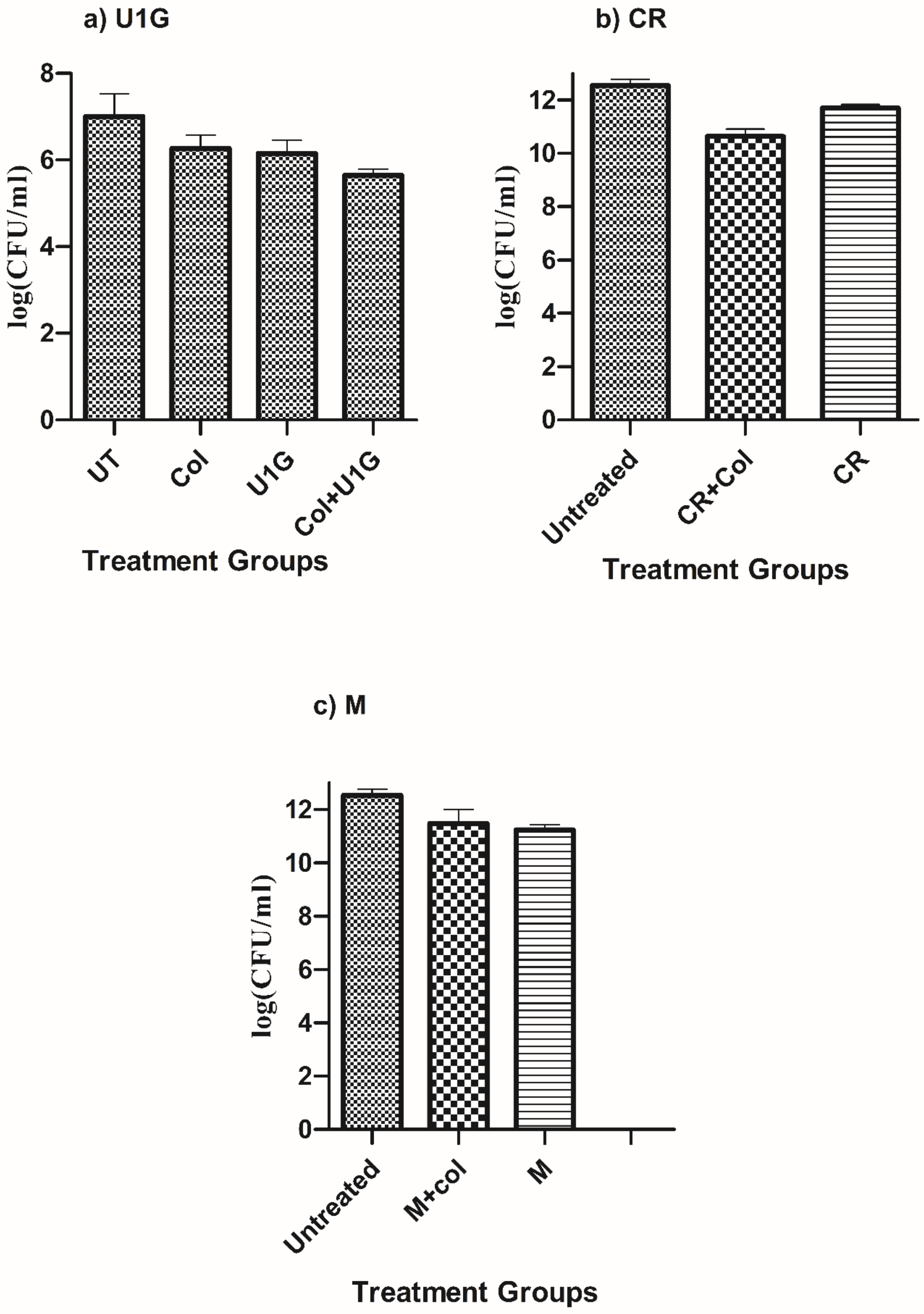

Table S1: Antimicrobial Resistant Genes in Genome of *E. coli* Clinical isolate U1007 using ResFinder.

**Antimicrobial**	**Class**	**Genetic Background**
amikacin	aminoglycoside	aac(6')-Ib-cr (aac(6')-Ib-cr_DQ303918), rmtB (rmtB_AB103506)
tobramycin	aminoglycoside	aac(6')-Ib-cr (aac(6')-Ib-cr_DQ303918), rmtB (rmtB_AB103506)
cefepime	beta-lactam	blaOXA-1 (blaOXA-1_HQ170510), blaNDM-5 (blaNDM-5_JN104597), blaCTX-M-15 (blaCTX-M-15_AY044436)
chloramphenicol	amphenicol	catB3 (catB3_U13880), catB3 (catB3_AJ009818)
piperacillin+tazobactam	beta-lactam	blaOXA-1 (blaOXA-1_HQ170510), blaNDM-5 (blaNDM-5_JN104597)
cefoxitin	beta-lactam	blaNDM-5 (blaNDM-5_JN104597)
ampicillin	beta-lactam	blaOXA-1 (blaOXA-1_HQ170510), blaTEM-1B (blaTEM-1B_AY458016), blaNDM-5 (blaNDM-5_JN104597), blaCTX-M-15 (blaCTX-M-15_AY044436)
ampicillin+clavulanic acid	beta-lactam	blaOXA-1 (blaOXA-1_HQ170510), blaNDM-5 (blaNDM-5_JN104597)
cefotaxime	beta-lactam	blaNDM-5 (blaNDM-5_JN104597), blaCTX-M-15 (blaCTX-M-15_AY044436)
ciprofloxacin	quinolone	aac(6')-Ib-cr (aac(6')-Ib-cr_DQ303918) gyrA (p.S83L)
sulfamethoxazole	folate pathway antagonist	sul1 (sul1_U12338), sul2 (sul2_AY034138)
imipenem	beta-lactam	blaNDM-5 (blaNDM-5_JN104597)
trimethoprim	folate pathway antagonist	dfrA17 (dfrA17_FJ460238)
nalidixic acid	quinolone	gyrA (p.S83L), gyrA (p.D87N)
ertapenem	beta-lactam	blaNDM-5 (blaNDM-5_JN104597)
tetracycline	tetracycline	tet(A) (tet(A)_AJ517790)
ceftazidime	beta-lactam	blaNDM-5 (blaNDM-5_JN104597), blaCTX-M-15 (blaCTX-M-15_AY044436)
temocillin	beta-lactam	blaNDM-5 (blaNDM-5_JN104597)
gentamicin	aminoglycoside	rmtB (rmtB_AB103506)
meropenem	beta-lactam	blaNDM-5 (blaNDM-5_JN104597)
azithromycin	macrolide	mph(A) (mph(A)_D16251)
isepamicin	aminoglycoside	rmtB (rmtB_AB103506)
hydrogen peroxide	peroxide	sitABCD (sitABCD_AY598030)
fluoroquinolone	quinolone	aac(6')-Ib-cr (aac(6')-Ib-cr_DQ303918)
cephalothin	beta-lactam	blaTEM-1B (blaTEM-1B_AY458016)
amoxicillin+clavulanic acid	beta-lactam	blaOXA-1 (blaOXA-1_HQ170510), blaNDM-5 (blaNDM-5_JN104597)
erythromycin	macrolide	mph(A) (mph(A)_D16251)
kanamycin	aminoglycoside	rmtB (rmtB_AB103506)

piperacillin	beta-lactam	blaOXA-1 (blaOXA-1_HQ170510), blaTEM-1B (blaTEM-1B_AY458016), blaNDM-5 (blaNDM-5_JN104597), blaCTX-M-15 (blaCTX-M-15_AY044436)
amoxicillin	beta-lactam	blaOXA-1 (blaOXA-1_HQ170510), blaTEM-1B (blaTEM-1B_AY458016), blaNDM-5 (blaNDM-5_JN104597), blaCTX-M-15 (blaCTX-M-15_AY044436)
ethidium bromide	quaternary ammonium compound	qacE (qacE_X68232)
sisomicin	aminoglycoside	aac(6')-Ib-cr (aac(6')-Ib-cr_DQ303918), rmtB (rmtB_AB103506)
spectinomycin	aminocyclitol	aadA5 (aadA5_AF137361)
doxycycline	tetracycline	tet(A) (tet(A)_AJ517790)
arbekacin	aminoglycoside	rmtB (rmtB_AB103506)
cefixime	beta-lactam	blaNDM-5 (blaNDM-5_JN104597)
benzylkonium chloride	quaternary ammonium compound	qacE (qacE_X68232)
ceftriaxone	beta-lactam	blaCTX-M-15 (blaCTX-M-15_AY044436)
telithromycin	macrolide	mph(A) (mph(A)_D16251)
ticarcillin	beta-lactam	blaTEM-1B (blaTEM-1B_AY458016), blaCTX-M-15 (blaCTX-M-15_AY044436)
chlorhexidine	quaternary ammonium compound	qacE (qacE_X68232)
cetylpyridinium chloride	quaternary ammonium compound	qacE (qacE_X68232)
spiramycin	macrolide	mph(A) (mph(A)_D16251)
dibekacin	aminoglycoside	aac(6')-Ib-cr (aac(6')-Ib-cr_DQ303918)
netilmicin	aminoglycoside	aac(6')-Ib-cr (aac(6')-Ib-cr_DQ303918)
aztreonam	beta-lactam	blaCTX-M-15 (blaCTX-M-15_AY044436)
streptomycin	aminoglycoside	aadA5 (aadA5_AF137361), aph(3")-Ib (aph(3")-Ib_AF321551), aph(6)-Id (aph(6)-Id_CP000971)
colistin *	polymyxin	emrA_2, emrB_2

* genes identified using Roary.

Table S2: Antimicrobial profile of *E. coli* clinical isolate U1007 determined by two fold microbroth dilution assay

Class	Antibiotics	MIC(μg/mL)
Cephalosporin	Ceftriaxone	>128
Carbapenem	Meropenem	>128
Fluoroquinolones	Ciprofloxacin	>128
	Levofloxacin	32
	Norfloxacin	>128
Aminoglycosides	Gentamicin	>128
	Tobramycin	>128
	Streptomycin	128
	Kanamycin	>128
Antimycobacterial	Rifampicin	32
Macrolides	Erythromycin	>128
Tetracycline	Minocycline	8
Polymyxin	Colistin	4

Table S3: Features of U1G, genome annotated by RAST

Start	Stop	RAST
170	493	Phage protein
584	943	hypothetical protein
997	1203	hypothetical protein
1330	1713	Phage integrase
1857	2156	hypothetical protein
2159	2377	hypothetical protein
2374	2547	hypothetical protein
2571	3044	Phage HNH homing endonuclease (ACLAME 27)
3049	3180	hypothetical protein
3314	3622	hypothetical protein
3619	3840	hypothetical protein
3837	4094	hypothetical protein
4091	4411	Phage antirepressor protein
4414	4596	hypothetical protein
4593	4973	hypothetical protein
5027	5845	hypothetical protein
5835	5996	hypothetical protein
6043	7260	hypothetical protein
7318	7500	hypothetical protein
7575	8408	Phage fibritin (wac) protein
8474	8788	Superinfection exclusion protein (Protein gp17)
8893	9081	hypothetical protein
9078	9338	hypothetical protein
9335	9520	hypothetical protein
9521	9847	hypothetical protein
9882	10202	Phage protein
10313	10882	HNH homing endonuclease # Phage intron
11142	11816	Phage protein
11824	12327	hypothetical protein
12324	12998	hypothetical protein
12998	13504	Phage protein

13514	13723	hypothetical protein
13797	14039	hypothetical protein
14055	14387	hypothetical protein
14554	14997	hypothetical protein
14997	15941	Phage protein
16009	16221	hypothetical protein
16214	16543	Phage protein
16536	16730	hypothetical protein
16774	19341	Phage rIIA lysis inhibitor
19346	21406	hypothetical protein
21469	21864	hypothetical protein
21911	22045	hypothetical protein
22108	22233	Phage protein
22725	24077	Phage DNA helicase
24088	24618	hypothetical protein
24628	26442	DNA polymerase I (EC 2.7.7.7), phage-associated
26439	27095	Phage protein (ACLAME 141)
27076	28047	DNA polymerase I (EC 2.7.7.7), phage-associated
28044	28349	hypothetical protein
28349	28822	hypothetical protein
28822	29799	hypothetical protein
29796	30233	Phage-associated homing endonuclease
30276	32426	Phage-associated DNA primase
32483	32884	Phage protein
32950	33234	Phage protein
33430	34071	hypothetical protein
34071	34625	hypothetical protein
34627	35067	hypothetical protein
35299	35412	hypothetical protein
36836	37216	hypothetical protein
48080	37326	DNA polymerase, phage-associated
49890	48175	hypothetical protein
50138	49884	hypothetical protein
50594	50151	hypothetical protein

53262	50608	hypothetical protein
54100	53264	hypothetical protein
54809	54177	hypothetical protein
56085	54883	hypothetical protein
57322	56102	Phage tape measure protein
57686	57342	Phage protein
59970	57700	Phage portal (connector) protein
60479	59973	hypothetical protein
60891	60460	Phage protein
61094	60996	hypothetical protein
61657	61328	Phage tail length tape-measure protein T
62449	61916	hypothetical protein
63285	62485	hypothetical protein
63800	63477	Phage tailspike protein
65447	64050	hypothetical protein
67642	65492	hypothetical protein
68349	67639	hypothetical protein
69945	68356	Phage terminase, large subunit
70627	69938	hypothetical protein
70811	71089	hypothetical protein
71654	71968	hypothetical protein
71972	72292	hypothetical protein
72292	72576	hypothetical protein
72573	72887	hypothetical protein
72877	73215	hypothetical protein

Table S4: Features of CR phage genome annotated by RAST

start	stop	strand	function
229	351	+	hypothetical protein
433	819	+	Phage protein
1010	1711	+	Phage protein
1714	1824	+	Phage major capsid protein of Caudovirales
1977	2762	+	Phage major capsid protein of Caudovirales
2824	3165	+	Phage fibritin (wac) protein
3202	3381	+	hypothetical protein
3385	3537	+	hypothetical protein
3531	3758	+	Phage protein (ACLAME 313)
3761	4039	+	Phage protein
4042	4656	+	Phage protein
4656	4787	+	hypothetical protein
4784	5143	+	Phage protein
5140	5640	+	Phage protein
5640	6053	+	Phage protein
6056	7222	+	Phage protein
7804	7250	-	hypothetical protein
8289	7804	-	3'-phosphatase, 5'-polynucleotide kinase, phage-associated
8975	8286	-	hypothetical protein
9466	8972	-	Phage protein
9807	9481	-	hypothetical protein
9972	10388	+	Phage protein
10487	10750	+	hypothetical protein
10743	12587	+	Phage tail tape measure
12692	13054	+	Phage tail tape measure
13054	14001	+	Phage protein
14133	14444	+	hypothetical protein
14448	14963	+	Phage protein
14960	15325	+	Phage protein
15388	17874	+	Phage protein
17887	19332	+	Phage tailspike
19345	19461	+	hypothetical protein
19533	19721	+	hypothetical protein
19887	19750	-	Phage protein
20399	19884	-	hypothetical protein
21820	20396	-	Phage DNA helicase
22501	22310	-	Phage protein
22831	22532	-	Phage protein
23006	22812	-	hypothetical protein
23220	23089	-	hypothetical protein
23614	23210	-	DNA polymerase I (EC 2.7.7.7), phage-associated
23976	23659	-	DNA polymerase I (EC 2.7.7.7), phage-associated

25409	24006	-	DNA polymerase I (EC 2.7.7.7), phage-associated
26095	25469	-	Phage protein
26430	26179	-	hypothetical protein
27533	26427	-	Phage protein
27667	27527	-	Phage protein
28011	27664	-	Phage protein
28280	28008	-	hypothetical protein
28591	28277	-	hypothetical protein
29153	28635	-	13.88 kDa late protein
29403	29176	-	hypothetical protein
29526	29741	+	hypothetical protein
30390	29758	-	Phage replicative DNA helicase, repA
30635	30381	-	hypothetical protein
32014	30644	-	Phage replicative DNA helicase, repA
32235	32074	-	Phage protein
32473	32303	-	Phage protein
32940	32590	-	hypothetical protein
32971	33189	+	hypothetical protein
33208	33393	+	hypothetical protein
33393	33632	+	hypothetical protein
33899	34090	+	hypothetical protein
34094	34285	+	hypothetical protein
34289	34432	+	hypothetical protein
34436	34636	+	hypothetical protein
34772	35002	+	hypothetical protein
35072	35449	+	hypothetical protein
35446	35649	+	Phage protein
35652	35855	+	hypothetical protein
35855	36148	+	hypothetical protein
36145	36585	+	Phage protein
36645	36953	+	hypothetical protein
36946	37218	+	gp55
37196	37681	+	Phage lysin (EC 3.2.1.17)
38028	38177	+	Phage protein
38174	38371	+	hypothetical protein
38368	38523	+	Phage protein
38520	38717	+	hypothetical protein
38698	38886	+	hypothetical protein
38974	39141	+	hypothetical protein
39313	39161	-	hypothetical protein
39332	39568	+	Phage protein
39565	39714	+	hypothetical protein
39841	40386	+	Phage protein
40383	40832	+	Putative phage terminase
40919	41413	+	Phage terminase, large subunit
41416	41631	+	Phage terminase, large subunit
41644	42798	+	62kDa structural protein

42774	43112	+	62kDa structural protein
43427	43146	-	hypothetical protein
43536	44288	+	Putative head protein (ACLAME 50)
44435	44578	+	Putative head protein (ACLAME 50)
44581	45039	+	Phage fibritin (wac) protein
45235	45068	-	hypothetical protein

Table S5: Features of M phage genome annotated by RAST

start	stop	strand	function
1	180	+	hypothetical protein
352	200	-	hypothetical protein
371	607	+	Phage protein
604	753	+	hypothetical protein
930	1046	+	hypothetical protein
1080	1427	+	Phage protein
1424	2017	+	Phage terminase
2383	2676	+	Phage terminase, large subunit
2689	3357	+	62kDa structural protein
3350	4159	+	62kDa structural protein
4475	4194	-	hypothetical protein
4584	5474	+	Phage protein
5488	5631	+	Putative head protein (ACLAME 50)
5714	6091	+	Phage fibritin (wac) protein
6088	6192	+	hypothetical protein
6398	6514	+	hypothetical protein
6792	6574	-	hypothetical protein
7191	7892	+	Phage protein
7895	8347	+	Phage major capsid protein of Caudovirales
8344	8946	+	Phage major capsid protein of Caudovirales
9009	9128	+	hypothetical protein
9148	9351	+	Phage fibritin (wac) protein
9389	9568	+	hypothetical protein
9572	9724	+	hypothetical protein
9718	10227	+	Phage protein
10230	10844	+	Phage protein
10844	10975	+	hypothetical protein
10972	11331	+	Phage protein
11328	11828	+	Phage protein
11828	12241	+	Phage protein
12244	13410	+	Phage protein
13993	13439	-	hypothetical protein
14478	13993	-	3'-phosphatase, 5'-polynucleotide kinase, phage-associated
14753	14475	-	Phage transcriptional regulator
15608	14754	-	Phage protein
16000	15674	-	hypothetical protein
16165	16581	+	Phage protein
16680	16943	+	hypothetical protein
16936	19248	+	Phage tail tape measure
19285	19941	+	Phage protein
20330	20641	+	hypothetical protein
20645	21160	+	Phage protein
21157	21522	+	Phage protein
21513	24071	+	Phage protein
24084	24416	+	Phage tailspike

24422	25735	+	hypothetical protein
25732	25920	+	hypothetical protein
26086	25949	-	Phage protein
26238	26083	-	hypothetical protein
26600	26319	-	hypothetical protein
26779	26597	-	Phage DNA helicase
27005	26889	-	Phage DNA helicase
28023	26965	-	Phage DNA helicase
28516	28397	-	Phage-associated homing endonuclease
28704	28513	-	Phage protein
29034	28735	-	Phage protein
29209	29015	-	hypothetical protein
29424	29293	-	hypothetical protein
29603	29418	-	DNA polymerase I (EC 2.7.7.7), phage-associated
29830	29600	-	DNA polymerase I (EC 2.7.7.7), phage-associated
30194	29868	-	DNA polymerase I (EC 2.7.7.7), phage-associated
30352	30224	-	DNA polymerase I (EC 2.7.7.7), phage-associated
31274	30378	-	DNA polymerase I (EC 2.7.7.7), phage-associated
31628	31455	-	DNA polymerase I (EC 2.7.7.7), phage-associated
32315	31689	-	Phage protein
32654	32436	-	hypothetical protein
32989	32651	-	Phage protein
33404	33051	-	Phage protein
33663	33472	-	Phage protein
33830	33684	-	Phage protein
34029	33895	-	Phage protein
34243	34040	-	Phage protein
34512	34240	-	hypothetical protein
34823	34509	-	hypothetical protein
35087	34866	-	13.88 kDa late protein
35356	35114	-	hypothetical protein
35540	35391	-	hypothetical protein
35647	35862	+	hypothetical protein
36512	35880	-	Phage replicative DNA helicase, repA
38122	36503	-	Phage replicative DNA helicase, repA
38342	38181	-	Phage protein
38580	38410	-	Phage protein
39038	38751	-	hypothetical protein
39094	39312	+	hypothetical protein
39331	39516	+	hypothetical protein
39857	39985	+	hypothetical protein
40048	40239	+	hypothetical protein

40243	40434	+	hypothetical protein
40438	40581	+	hypothetical protein
40585	40785	+	hypothetical protein
40921	41151	+	hypothetical protein
41218	41754	+	hypothetical protein
41751	41954	+	Phage protein
41955	42350	+	hypothetical protein
42595	42909	+	hypothetical protein
42969	43274	+	hypothetical protein
43271	43543	+	hypothetical protein
43580	43966	+	Phage lysin (EC 3.2.1.17)
44006	44122	+	hypothetical protein
44446	44595	+	Phage protein
44592	44789	+	hypothetical protein
44786	44941	+	Phage protein
44938	45135	+	hypothetical protein
45116	45292	+	hypothetical protein

Table S6: Receptor Binding proteins hits present in the bacteriophage genomes identified using blast search with RBP database. Top hits are reported in the table

Phage	RBP	Homologs identified by blast search	% similarity	E-value
U1G phage	Phage tailspike protein (107 aa)	Tail fiber protein of Salmonella phage SP1	85	4e-34
CR phage	Phage protein (828 aa)	Putative tail protein of Escherichia phage ST2	99	0.0
	Phage tailspike protein (481 aa)	Tailspike protein Salmonella phage FSL SP-049	81	9e-69
M phage	Phage protein (852 aa)	Putative tail protein of Escherichia phage ST2	99	0.0
	Phage tailspike protein (110 aa)	Tailspike protein of Salmonella phage FSL SP-049	86%	1e-56
	Hypothetical protein (437 aa)	tail fiber protein_Acinetobacter phage Petty	45%	1e-16
